# Adverse kidney related events following targeted therapies in lung cancer: a systematic review and network meta-analysis of randomized controlled trials

**DOI:** 10.3389/fphar.2025.1511171

**Published:** 2025-03-13

**Authors:** Song Ren, Wei Wang, Xiaoxiu Yao, Wenyan Fang, Guisen Li, Yunlin Feng, Min Xia

**Affiliations:** ^1^ Department of Nephrology and Institute of Nephrology, Sichuan Academy of Medical Sciences and Sichuan Provincial People’s Hospital, School of Medicine, University of Electronic Science and Technology of China, Sichuan Clinical Research Centre for Kidney Diseases, Chengdu, China; ^2^ Department of Health Management, Damian Honghe Community Health Service Center of Longquanyi District, Chengdu, China; ^3^ Department of Nephrology, Zhongjiang County People’s Hospital, Deyang, China; ^4^ Cancer Center, Sichuan Academy of Medical Sciences and Sichuan Provincial People’s Hospital, University of Electronic Science and Technology of China, Chengdu, China

**Keywords:** renal dysfunction, proteinuria, urinary tract infection, electrolyte disorder, targeted therapy, lung cancer, meta-analysis

## Abstract

**Background:**

To summarize current evidence on kidney related adverse events (AEs) following targeted therapies in lung cancer from trial settings.

**Methods:**

A systematic search was conducted in MEDLINE, EMBASE, and Cochrane Central Library. Randomized controlled trials that had reported kidney related AEs following targeted therapies in lung cancer were eligible. Outcomes included renal dysfunction as reported, increased serum creatinine, proteinuria, urinary tract infection (UTI), and electrolyte disorders. The risk of bias was assessed using the Cochrane guidelines. The incidence of the examined outcomes, along with their corresponding 95% confidence intervals (CIs), were combined using a random-effects model. Network analysis was applied if the comparisons had passed the consistency test. Publication bias was assessed using Funnel plot analysis.

**Results:**

57 studies encompassing 11,497 patients were included. The pooled incidences (95% CI) of acute kidney injury (AKI), increased serum creatinine, proteinuria, and UTI following targeted therapies in lung cancer were 1% (0%, 2%), 4% (1%, 8%), 9% (6%, 13%), and 6% (2%, 12%), respectively. Targeted therapies did not increase the risk of AKI, yet were associated with higher incidence of proteinuria, particularly vascular endothelial growth factor inhibitors containing therapies. Multiple electrolyte disorders could be observed following targeted treatments, with the pooled incidences ranging from 4% to 21%; however, most electrolytes disorders had limited number of reports. Most of the reported kidney related AEs were of Common Terminology Criteria for Adverse Events (CTCAE) grade 1 or 2. Publication bias was present for kidney related AEs excluding AKI.

**Conclusion:**

Kidney related adverse events are not uncommon following targeted therapies in lung cancer in trial settings. In comparison to chemotherapy alone, targeted therapies did not increase the risk of AKI, yet were associated with higher risk of proteinuria. Proteinuria and electrolytes disorders are more often observed than renal dysfunction and UTI. All types of AEs were mostly mild in severity.

**Systematic Review Registration:**

PROSPERO CRD42023441979.

## Introduction

Lung cancer accounts for 11.6% of all cancers globally and is the reason of approximately one-fifths of the cancer related deaths (Sung et al., 2021). Early targeted therapies provide hope for advances in the treatment of lung cancer, particularly for patients with surgically unresectable lesions or distant metastasis (Hirsch et al., 2017; Lahiri et al., 2023; Miller and Hanna, 2021). However, early and intensive targeted therapies are associated with adverse events (AEs) in multiple organs, among which the most reported are gastrointestinal symptoms such as diarrhea, vomit, and nausea, as well as skin reactions including rash, acne, or injection site reaction (Ruiz et al., 2014).

Notably, adverse kidney related outcomes also occur subsequent to targeted therapies, sometimes even causing delay or suspension in the anti-cancer treatments. Kidney related AEs following targeted therapies include impaired renal function evidenced by elevated serum creatinine, proteinuria, urinary tract infection, and electrolyte disorders (Ruiz et al., 2014). We have even observed a few cases who even advanced to dialysis-dependent stage after targeted therapies in our own practice. Understanding the profile of kidney related AEs following targeted therapies help communications between patients and healthcare professionals for clinical decision making and might aid to reduce the chance of AEs in high-risk patients. However, the incidence, severity and outcomes of kidney related AEs are only sporadically reported in individual clinical trials, lacking a summary of existing evidence in this field.

Therefore, we conducted this systematic review and meta-analysis to elucidate the incidences of unfavorable kidney related outcomes following targeted therapies in lung cancer patients, to enhance the understanding of this subject and provide references for clinical practice.

## Materials and methods

### Data sources and searches

We conducted a comprehensive search identify eligible studies published until 19 August 2023 in EMBASE via Ovid, Cochrane Central Library via Ovid, and MEDLINE via PubMed, adhering to the guidelines outlined in the Preferred Reporting Items for Systematic Review and Meta-Analyses (PRISMA) statement (Liberati et al., 2009). The search terms included appropriate text terms related to the names and targeted molecules of commercially available pharmaceuticals for targeted therapies in lung cancers, randomized controlled trial, and lung cancer (Supplementary Table 1). No restrictions were imposed on publication date or language. The systematic review was prospectively registered on PROSPERO (Identifier# CRD42023441979).

### Study selection

Eligible studies were randomized controlled trial (RCTs) that had reported pairwise comparison among different targeted therapies, combination of targeted therapies and conventional chemotherapy, and chemotherapy alone in lung cancers as well as kidney related AEs following treatments. Only studies in adults were considered. No restriction was applied to the category of targeted agents or targeted molecules of treatment.

Two reviewers (S.R. and W.W.) independently conducted the screening process using a standardized approach. The titles and abstracts of all retrieved records from the database search were carefully examined. Duplicates, pediatric studies, reviews, editorials, commentaries, case reports, study protocols, conference abstracts lacking sufficient information, non-human studies, studies irrelevant to lung cancer, non-RCT studies, secondary analysis or pooled analysis of RCT trials, studies that had not reported kidney related outcomes or any targeted therapy, and studies that had not compared different categories of pharmaceutical treatment were excluded. Additionally, the reference lists of included studies or important reviews were also reviewed to identify any relevant studies. Any discrepancy was adjudicated by a third reviewer (Y.L.F.).

### Outcomes

All kidney related AEs were considered as study outcomes in this systematic review and were classified into four categories. The first category was renal dysfunction, including the diagnosis of acute kidney injury (AKI) or acute kidney failure and increased serum creatinine evidenced by laboratory examinations. The second category was proteinuria as indicated by abnormal urine findings. The third category was urinary tract infection (UTI). The fourth category was electrolytes disorders, including abnormalities in serum levels of sodium, potassium, calcium, phosphorus, and magnesium.

All adverse events were recorded as per the reporting in individual studies and quantified using their respective incidences reported in the corresponding studies.

### Data extraction and quality assessment

The extracted data included authors’ names, publication year, geographical location, total sample size of the study population, details of treatments employed, number of patients in both control and interventional groups, numbers of study outcomes in both control and interventional groups, and the Common Terminology Criteria for Adverse Events (CTCAE) grade of the adverse events (if reported). Treatments were classified into conventional chemotherapy and different categories of targeted therapies based on the targeted molecules.

Two reviewers (S.R. and W.W.) independently extracted data from included studies and compiled them into a shared document. Any discrepancy was resolved by the third reviewer (Y.L.F.).

### Critical appraisal

Two reviewers (S.R. and Y.L.F.) independently assessed the risk of bias of included studies based on the 7-item criteria in the RevMan analysis software provided by the Cochrane Collaboration (2022) (Higgins et al., 2019). Any discrepancy was resolved by consensus.

### Data synthesis and analysis

Data analysis and synthesis were performed using Stata (version 17.0; Stata Corporation, TX, United States) and Review Manager (RevMan 5.35) software.

The pooled occurrences of the examined outcomes, along with their corresponding 95% confidence intervals (CIs), were combined using a random-effects model, with each study group in the RCT studies treated as an independent arm. Additionally, the meta-analysis for study outcomes for which the assumption of consistency in the network analysis was verified using a design-by-treatment approach (Higgins et al., 2012) included direct comparisons for each pair of treatments and the network meta-analysis for multiple comparisons including indirect comparisons via pooled odds ratios (ORs) with 95% confidence intervals (CIs) using a random-effects model. Network map was used to shown the interactions among different treatments and the treatments were sorted in rank based on surface under the cumulative ranking curve (SUCRA) (Salanti et al., 2011) and graphically illustrated using the ranking panel plots. The higher the rank, the superior the treatment effect. Subgroup analyses were performed based on the category of treatments and the severity of adverse events evaluated by the CTCAE grade. Statistical heterogeneity was estimated using the I^2^ statistic, for which an I^2^ value of ≤25%, between 26% and 75%, and >75% represents low, moderate, and high heterogeneity, respectively (Ioannidis, 2008). A two-sided p value of <0.05 was considered statistically significant. Publication bias was assessed by visual inspection of funnel plots and comparison adjusted funnel plots.

## Results

### Search findings

**FIGURE 1:**
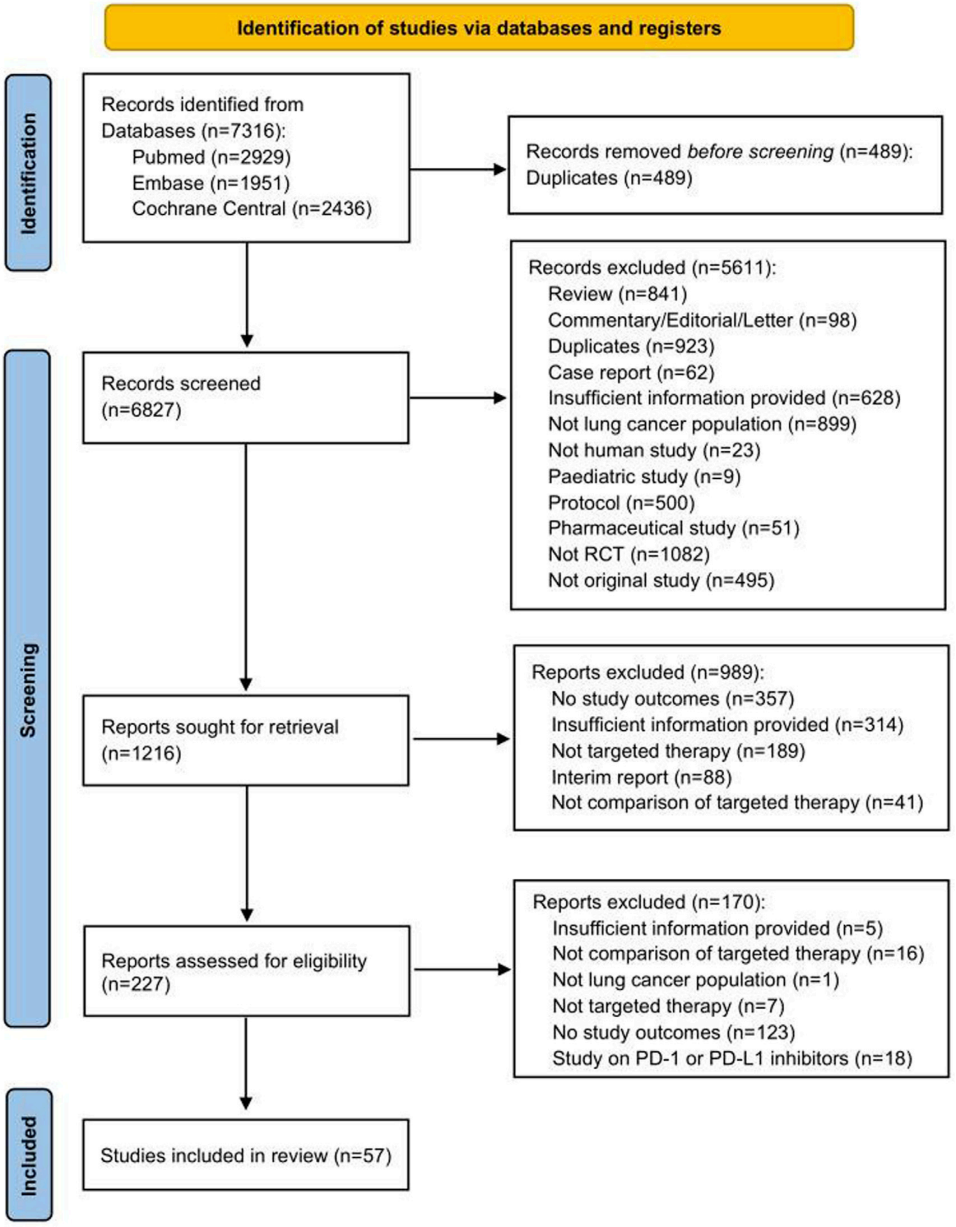
PRISMA flow chart for study selection. Abbreviations: RCT, randomized controlled trial.

A total of 6,827 records were initially identified through literature searching and after removing duplicates. 6,600 records were excluded after screening the titles and abstracts, and another 170 publications were further excluded after full text review. Finally, 57 RCT studies were included in this systematic review (see Figure 1).

### Study characteristics

57 RCT studies encompassing 11,497 patients were included in this systematic review (Ahn et al., 2012; Argiris et al., 2017; Ciuleanu et al., 2018; Ciuleanu et al., 2013; Crinò et al., 2008; Doebele et al., 2015; Du et al., 2013; Ellis et al., 2014; Gaafar et al., 2011; Garon et al., 2014; Goldman et al., 2020; Herbst et al., 2018; Johnson et al., 2013; Karayama et al., 2016; Kenmotsu et al., 2022; Lara et al., 2016; Leighl et al., 2017; Lynch et al., 2009; Miller et al., 2012; Nakagawa et al., 2019; Niho et al., 2012; Paz-Ares et al., 2015; Paz-Ares et al., 2017; Pérol et al., 2012; Pujol et al., 2015; Ramlau et al., 2012; Reck et al., 2016; Reck et al., 2009; Sandler et al., 2006; Scagliotti et al., 2012; Schuler et al., 2016; Sequist et al., 2011; Seto et al., 2014; Shaw et al., 2017; Shi et al., 2017; Socinski et al., 2010; Spigel et al., 2018; Spigel et al., 2013; Spigel et al., 2017a; Spigel et al., 2017b; Steendam et al., 2021; Stephenson et al., 2014; Stinchcombe et al., 2019; Tada et al., 2022; Takeda et al., 2010; Takeda et al., 2016; Thatcher et al., 2015; von Pawel et al., 2018; Wakelee et al., 2017a; Wakelee et al., 2017b; Witta et al., 2012; Wu et al., 2018; Wu et al., 2015; Yoh et al., 2016; Yoshioka et al., 2015; Zhong et al., 2018; Zhou et al., 2015). The majority of studies had been registered. The maximum follow-up duration was 108 months. Therapeutic regimens substantially varied among the studies, including combinations of two targeted therapies and chemotherapy, combination of one targeted therapy and chemotherapy, one or two targeted therapies, and chemotherapy alone. Detailed characteristics of the included studies are shown in Supplementary Table 2.

### Acute kidney injury following targeted treatments

**FIGURE 2:**
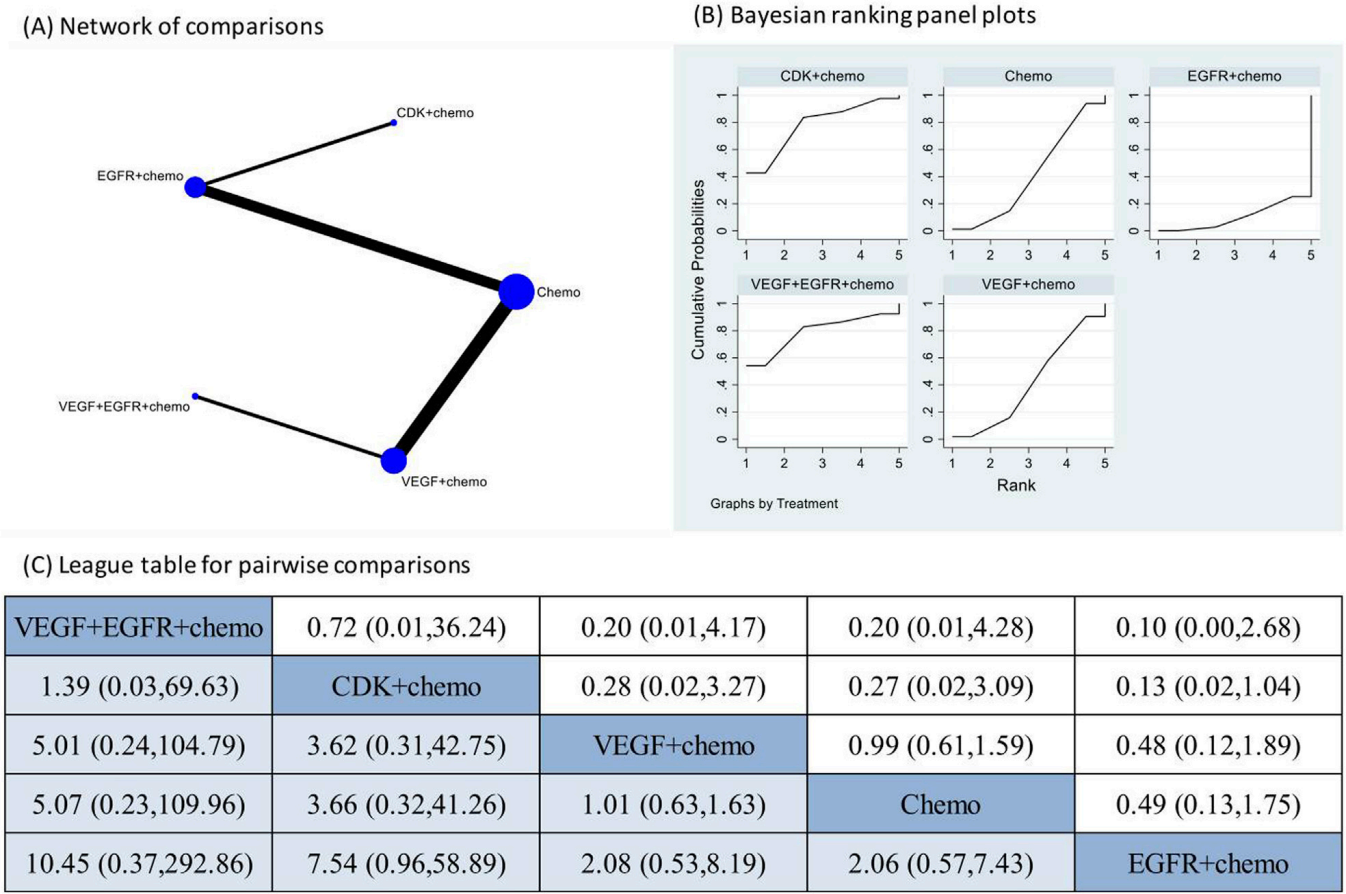
Network meta-analysis of AKI subsequent to the administration of targeted therapies in lung cancer. Note: **(A)** In the network of comparisons, the size of nodes is proportional to the total sample size of each treatment, and the width of lines is proportional to the number of studies in each pair of comparison. **(B)** Bayesian ranking panel plots indicate the higher the rank reflected by the area under curve, the superior the treatment to increase the risk of AKI. **(C)** The league table of pairwise comparison for the risk of AKI following treatments. All treatments are ordered based on AKI ranking.

The pooled incidence of AKI in the 10 RCT studies that had reported the occurrence of AKI following targeted therapies was 1% (95% CI: 0%, 2%) (Supplementary Figure 1). The treatment regimens encompassed one combination of two different targeted therapies and chemotherapy, three combinations of one targeted therapy and chemotherapy, and chemotherapy alone. The direct comparison of AKI following different treatment regimens did not reveal a significantly increased risk of AKI after any specific therapy (Supplementary Figure 2). Network meta-analysis was conducted after verification of consistency (Supplementary Table 3) and the results also indicated none of the four combination treatments significantly increased the risk of AKI compared to chemotherapy alone or each other (Figure 2). The subgroup analysis based on CTCAE grade indicated most of the AKI events were of CTCAE grade 1–2 (Supplementary Figure 3).

### Increased serum creatinine following targeted treatments

**FIGURE 3:**
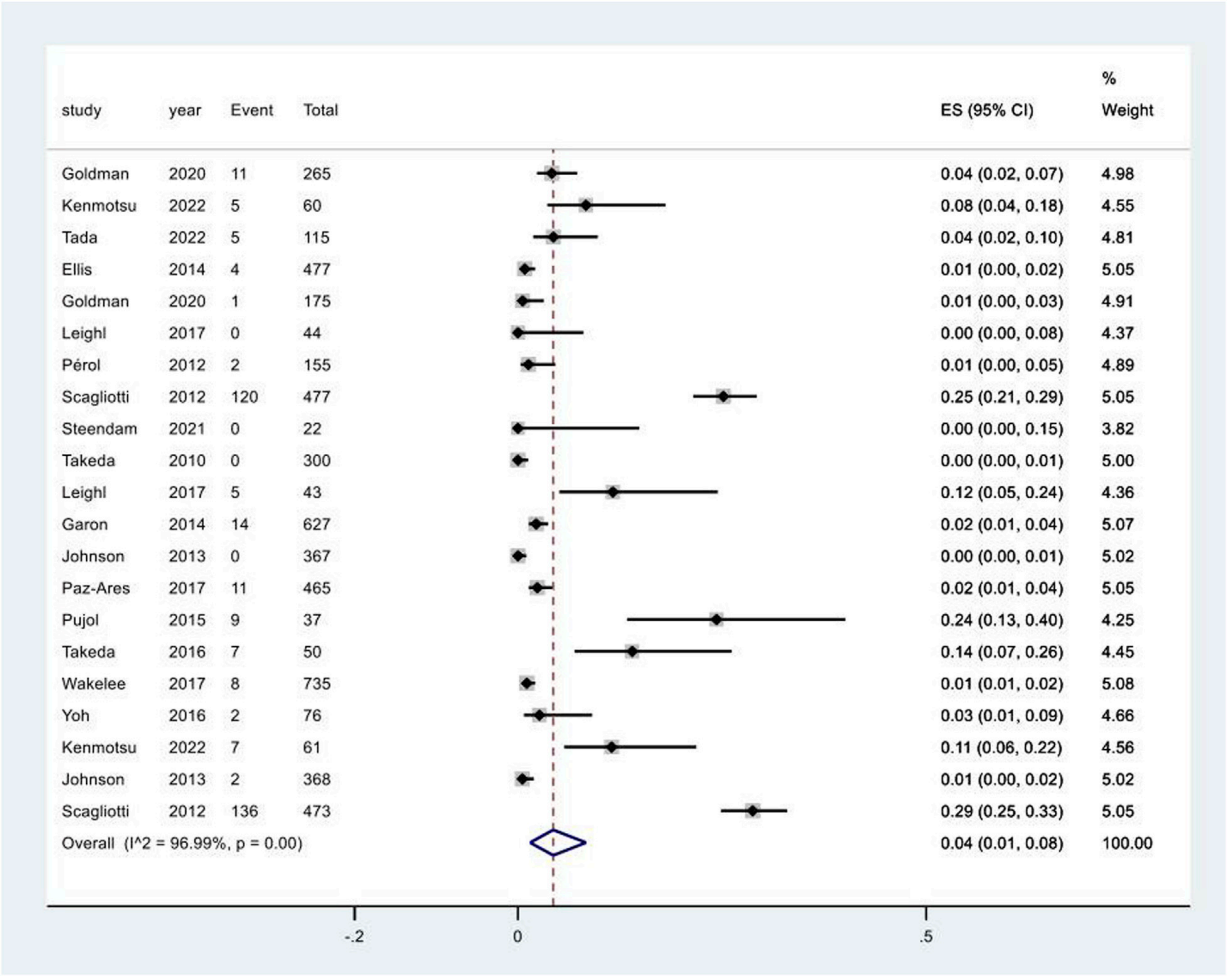
Aggregated occurrence rate of increased serum creatinine subsequent to the administration of targeted therapies in lung cancer. Notes: The pooled incidence of proteinuria was 5% (95% CI: 2%, 8%), with a high degree of heterogeneity observed among the studies (I^2^ = 96.3%, p < 0.01).

20 studies reported the occurrence of increased serum creatinine following targeted treatments. Network meta-analysis was not conducted due to the lack of verified consistency. The pooled incidence of increased serum creatinine following targeted treatments was 4% (95% CI: 1%, 8%), with a high degree of heterogeneity among the studies (I^2^ = 97.0%, p < 0.01) (Figure 3). Notably, the pooled incidence of increased serum creatinine in the chemotherapy groups in these studies was 4% (95% CI: 1%, 6%), having no significant difference compared to that following targeted therapies (p = 0.724) (Supplementary Figure 4). A further breaking down of targeted therapies revealed the pooled incidence of increased serum creatinine varied substantially across different targeted regimens, ranging from 2% (95% CI: 0%, 10%) in the combination of EGFR inhibitors and chemotherapy group to 12% (95% CI: 10%, 14%) in the combination of vascular endothelial growth factor receptor (VEGFR) and epidermal growth factor receptor (EGFR) inhibitors and chemotherapy group (Supplementary Figure 5). The pooled incidence of increased serum creatinine of CTCAE grade 1–2 was 6% (95% CI: 2%, 11%) (Supplementary Figure 6). Increased serum creatinine of CTCAE grade 3–4 was rarely observed (Supplementary Figure 7).

### Proteinuria following targeted treatments

**FIGURE 4:**
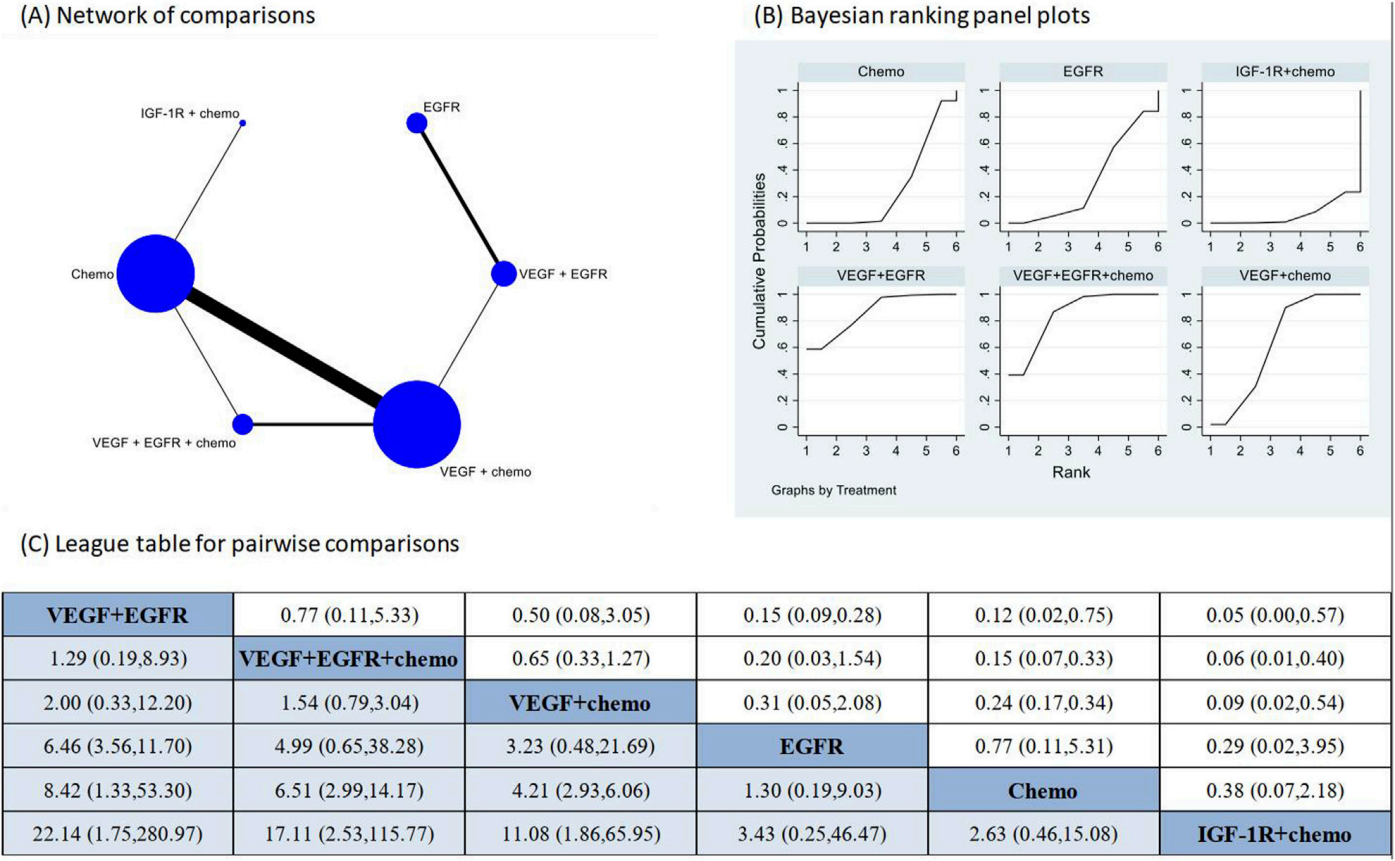
Network meta-analysis of proteinuria subsequent to the administration of targeted therapies in lung cancer. Note: **(A)** In the network of comparisons, the size of nodes is proportional to the total sample size of each treatment, and the width of lines is proportional to the number of studies in each pair of comparison. **(B)** Bayesian ranking panel plots indicate the higher the rank reflected by the area under curve, the superior the treatment to increase the risk of proteinuria. **(C)** The league table of pairwise comparison for the risk of proteinuria following treatments. All treatments are ordered based on proteinuria ranking. * Statistical significance.

The pooled incidence of proteinuria in the 23 studies that had reported this outcome was 9% (95% CI: 6%, 13%) (Supplementary Figure 8). The treatments examined in these studies included one combination of two targeted therapies and chemotherapy, two combinations of one targeted therapy and chemotherapy, one combination of two different targeted therapies, two targeted monotherapies, and chemotherapy alone. The direct comparison showed that three regimens that contained VEGFR inhibitors had significantly higher risk of proteinuria in comparison to chemotherapy alone (Supplementary Figure 9). Network meta-analysis was conducted after verification of consistency (Supplementary Table 4) and the results again revealed increased risk of proteinuria following the VEGFR inhibitors containing regimens in comparison to chemotherapy alone (Figure 4). The combination of IGF-1R targeted therapy and chemotherapy had the lowest risk of proteinuria. The subgroup analysis based on CTCAE grade indicated most of the proteinuria events were of CTCAE grade 1–2 (Supplementary Figure 10).

### Urinary tract infection following treatments

**FIGURE 5:**
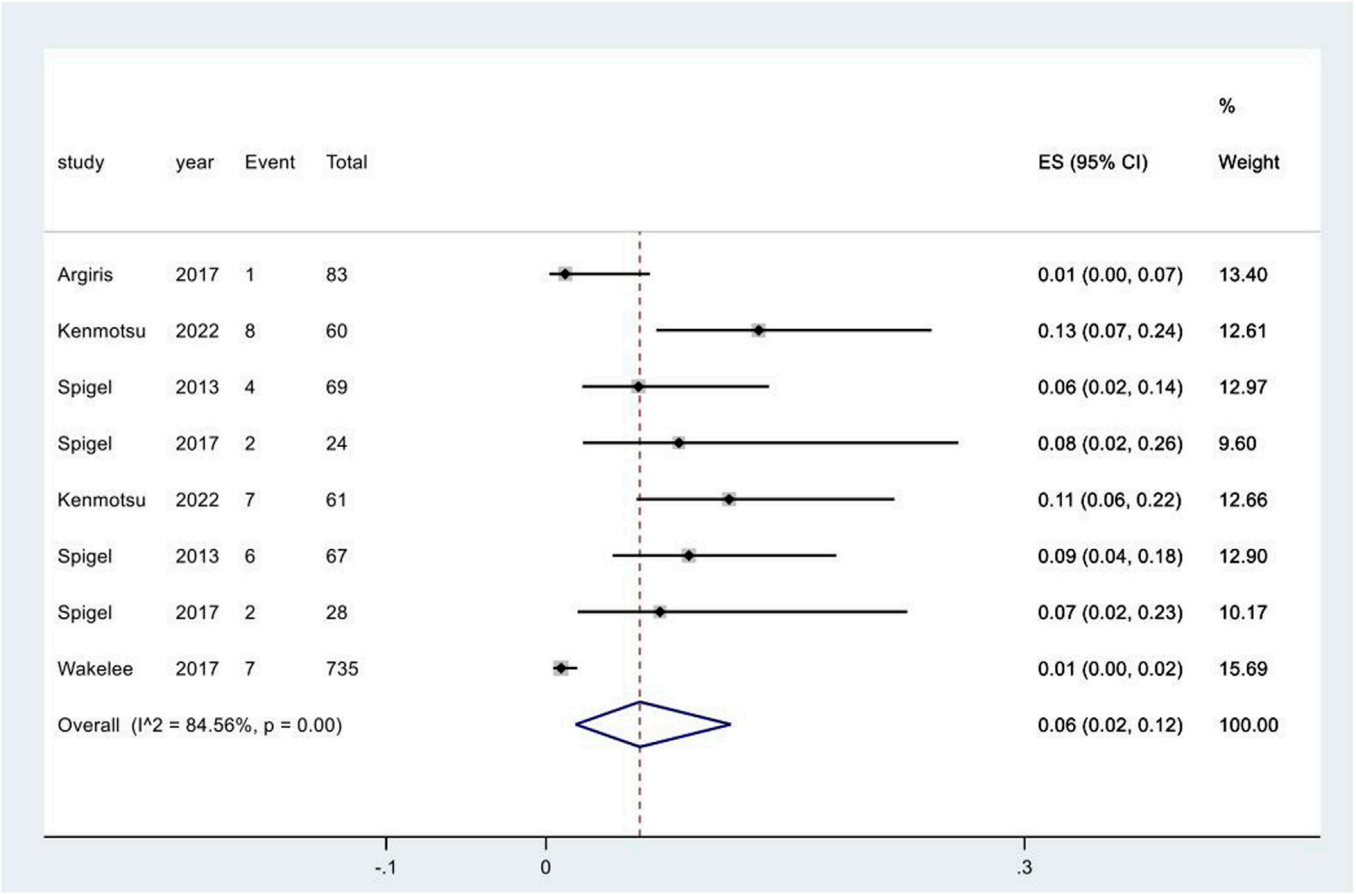
Urinary tract infection subsequent to the administration of targeted therapies in lung cancer. Notes: The pooled incidence of UTI was 7% (95% CI: 2%, 13%), with a high degree of heterogeneity observed among the studies (I^2^ = 91.7%, p < 0.01).

Five studies reported the adverse event of UTI following different regimens containing targeted therapies. The meta-analysis for UTI only included direct comparisons due to the limited number of studies. The pooled incidence was 6% (95% CI: 2%, 12%), with a high degree of heterogeneity observed among the studies (I^2^ = 84.6%, p < 0.01) (Figure 5). The pooled incidence of UTI in chemotherapy groups in these studies was 1% (95% CI: 0%, 1%), significantly lower than that following targeted therapy groups (p = 0.002) (Supplementary Figure 11).

### Electrolytes disorders following treatments

17 studies reported the adverse event of hypokalemia, of which the pooled incidence was 6% (95% CI: 3%, 9%) (Supplementary Figure 12). The pooled incidence of hypokalemia in the chemotherapy groups in these studies was 4% (95% CI: 2%, 6%), having no significant difference compared to that in the targeted therapy groups (p = 0.351) (Supplementary Figure 13). Analysis based on the types of targeted therapies indicated the pooled incidence of hypokalemia varied from 1% (95% CI: 0%, 3%) in the EGFR inhibitors group to 26% (95% CI: 23%, 29%) in the combination of VEGF and EGFR inhibitors and chemotherapy group (heterogeneity between groups: p < 0.01) (Supplementary Figure 14). The pooled incidence of hyperkalemia in the targeted therapy groups in four studies was 4% (95% CI: 1%, 8%) (Supplementary Figure 15), which was significantly higher than that in the chemotherapy groups in two studies (p = 0.037) (Supplementary Figure 16).

15 studies reported the adverse event of hyponatremia, of which the pooled incidence was 7% (95% CI: 4%, 11%) (Supplementary Figure 17). The pooled incidence of hyponatremia in the chemotherapy groups in these studies was 6% (95% CI: 3%, 11%), having no significant difference compared to that in the targeted therapy groups (p = 0.854) (Supplementary Figure 18).

The pooled incidence of hypocalcemia following targeted therapies in four studies was 12% (95% CI: 4%, 23%) (Supplementary Figure 19), which was significantly higher than that following chemotherapy alone reported in 22 studies (p < 0.01) (Supplementary Figure 20). The pooled incidence of hypercalcemia following targeted therapies in two studies was 4% (95% CI: 1%, 10%) (Supplementary Figure 21).

**FIGURE 6:**
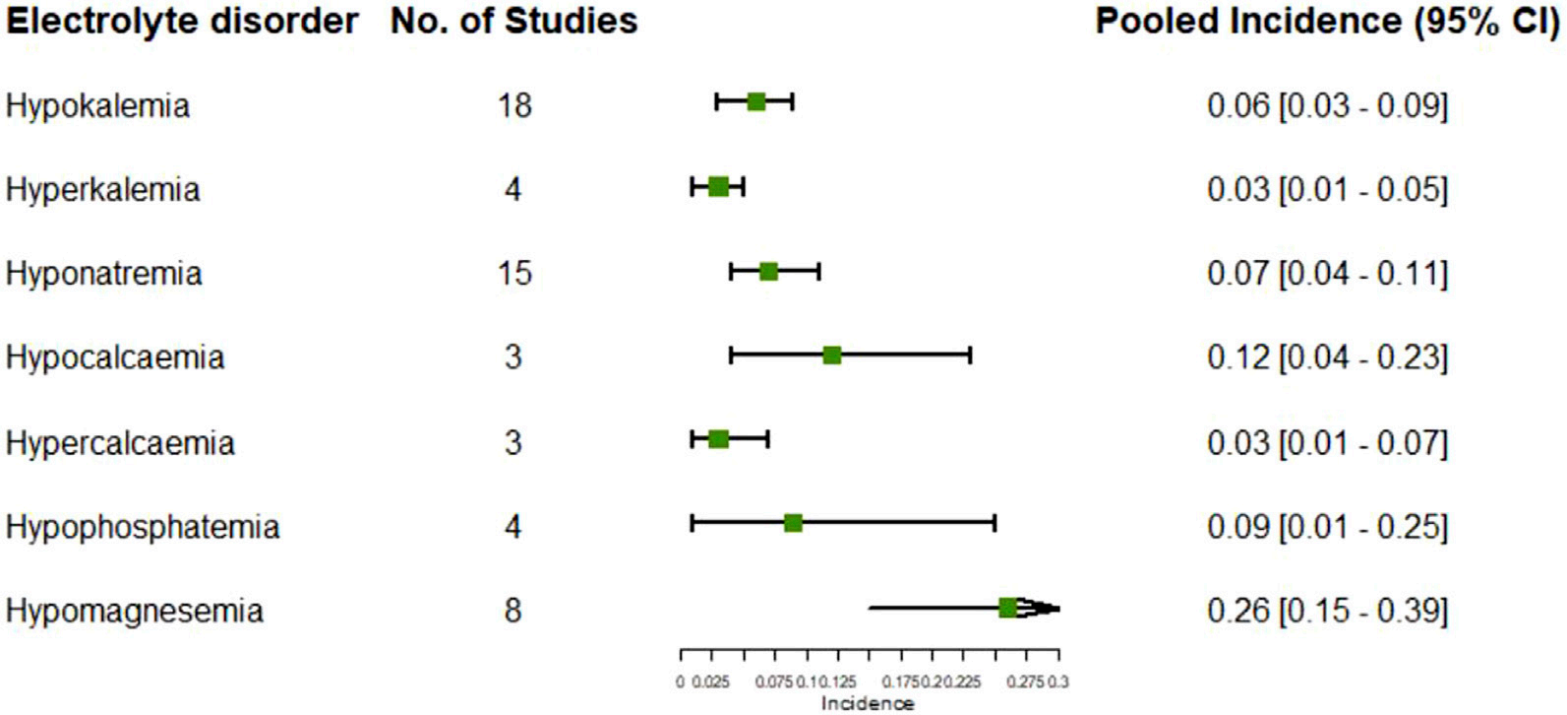
Pooled incidences of electrolytes disorders subsequent to the administration of targeted therapies in lung cancer.

Other reported electrolyte disorders following targeted therapies included hypophosphatemia reported in four studies and hypomagnesemia reported in eight studies, of which the pooled incidences were 12% (95% CI: 1%, 30%) (Supplementary Figure 22) and 26% (95% CI: 15%, 39%), respectively (Supplementary Figure 23). The summary of pooled incidences for all electrolytes disorders is shown in Figure 6.

### Risk of bias assessment

Critical appraisal indicated 16, 14, and 27 studies were rated as having low, high, and unclear risk of bias based on the 7-item Cochrane criteria (Supplementary Figure 24). The domain with the highest proportion of high risk was the performance bias (Supplementary Figure 25).

### Publication bias

Visual inspections of the funnel plot revealed absence of asymmetry for the outcome of AKI (Supplementary Figure 26); however, the presence of asymmetry was observed in the funnel plots for the outcomes of increased serum creatinine (Supplementary Figure 27), proteinuria (Supplementary Figure 28), UTI (Supplementary Figure 29), and electrolyte disorders (Supplementary Figure 30).

## Discussion

Our findings showed adverse kidney related outcomes are not uncommon following targeted therapies in lung cancer. The pooled incidences of AKI and proteinuria following targeted therapies in lung cancer were 1% (95% CI: 0%, 2%) and 7% (95% CI: 5%, 10%), respectively. The network meta-analysis indicated targeted therapies, either alone or in combination, did not increased the risk of AKI compared to chemotherapy alone, whereas VEGFR inhibitors containing therapies are associated with higher risk of proteinuria. The pooled incidences of increased serum creatinine and UTI following targeted therapies in lung cancer were 5% (95% CI: 2%, 8%) and 7% (95% CI: 2%, 13%), respectively, without significant differences in comparison with chemotherapy alone. Various electrolytes disorders could be observed following targeted treatments, with pooled incidences ranging from 4% to 21%; however, most electrolyte disorders had limited number of reports. Most of the reported events were of CTCAE grade 1–2.

Kidney injury following targeted treatment can manifests in various forms, among which renal function impairment, proteinuria, and electrolyte disorders are the most reported. The underlying histopathological diagnoses include thrombotic microangiography, focal segmental glomerulosclerosis, acute interstitial nephritis, and acute tubular necrosis (den Deurwaarder et al., 2012). Our findings that the pooled incidence of proteinuria was higher than that of renal function impairment reflected by the diagnosis of AKI or increased serum creatinine and also higher in VEGFR inhibitors containing therapies compared to chemotherapy alone are consistent with literature on glomerular injury caused by targeted agents, particularly VEGFR inhibitors (den Deurwaarder et al., 2012; Estrada et al., 2019; Izzedine et al., 2010). Notably, the pooled incidence of AKI in this study was lower than that associated with immune checkpoint inhibitors (ICIs) in non-small cell lung cancer (Zhu et al., 2022). Meanwhile, AKI and renal failure had been reported to top the ranking of kidney related AEs following ICIs treatment (Hu et al., 2021), again confirming a different toxicity spectrum compared with targeted therapies. Although proteinuria and electrolyte disorders were more often observed than AKI and UTI in this systematic review, proteinuria, UTI, and electrolyte disorders have gained far less attention than renal dysfunction in the literature, reflected by the fewer number of studies.

Our findings indicated all types of kidney related outcomes were minor in severity, necessitating no pharmaceutical interventions. These findings are consistent with our clinical observations. The timing of onset also matters. Therefore, close surveillance and regular examinations of renal function and urine analysis play a significant role in the follow up of lung cancer patients who are receiving targeted therapies, to monitor both the onset and outcomes of kidney related AEs. In addition, the assessment of renal function in these cases should be comprehensive, monitoring not only serum creatinine, but also urinary protein and electrolytes.

To our acknowledgment, this is the first systematic review and meta-analysis on the kidney related AEs following targeted therapies in lung cancer so far. This study benefited from a comprehensive literature search and unbiased comparisons of RCTs. In contrast to previous reports that focused on AKI or renal failure, this systematic review investigated the occurrence of all types of kidney related AEs, providing a full picture of relevant studies in this field. Future research on the mechanisms of nephrotoxicity caused by targeted agents will help to minimize the risk of adverse kidney related outcomes and improve patients’ survival.

There are still some limitations worth mentioning. First, the report bias should be considered when interpretate the results. Since the kidney related AEs are not the mainstay of adverse events following targeted therapies, they may be underestimated due to report bias. Second, the kidney related outcomes examined here were reported as adverse event without consensus definition, which might be an important source for the observed high heterogeneity. For example, for increased creatine, we were unable to know the increased amount or if the increased creatinine accounted for a diagnosis of AKI. Third, the chemotherapy was considered as a single group in the meta-analysis; however, the regimens substantially varied across different studies, from a single agent to combination of multiple agents, and the sequence of drug administration might also be different even for the same agents. Fourth, we cannot rule out the renal injury caused by lung cancer *per se* in this study. Although we tried to reduce the effect of this confounder by including RCT studies; however, network meta-analysis was only feasible for AKI and proteinuria. Fifth, this systematic review investigated the kidney related AEs in the clinical trial settings. Future studies and accumulative data from real-world cohorts will further help us gain more insight into this field.

## Conclusion

In summary, the findings suggested adverse kidney related outcomes are not uncommon following targeted therapies in lung cancer in trial settings. In comparison to chemotherapy alone, targeted therapies did not increase the risk of AKI, yet were associated with higher incidence of proteinuria, particularly VEGFR inhibitors containing therapies. Proteinuria and electrolytes disorders are more often observed than renal dysfunction and UTI. All types of AEs were mild in severity. Future studies and accumulative data from real-world cohorts will further help us to understand the whole picture.

## Data Availability

The original contributions presented in the study are included in the article/Supplementary Material, further inquiries can be directed to the corresponding authors.
