# Supplementary material for: Adverse kidney related events following targeted therapies in lung cancer: a systematic review and network meta-analysis of randomized controlled trials
Source: Front Pharmacol. 2025 Mar 13;16:1511171. doi: 10.3389/fphar.2025.1511171 (PMC11965906; doi:10.3389/fphar.2025.1511171)
Supplement: Supplementary file 1 [file DataSheet1.docx]

**Supplementary Materials**

[eTable 1. Search terms used in Embase via Ovid. 3](#_Toc166874206)

[eTable 2. General characteristics of studies included in meta-analysis. 5](#_Toc166874207)

[eTable 3. Test results of the consistency in the network analysis for acute kidney injury. 12](#_Toc166874208)

[eTable 4. Test results of the consistency in the network analysis for proteinuria. 13](#_Toc166874209)

[eFigure 1. Pooled incidence of AKI following regimens containing targeted therapy and chemotherapy alone. 14](#_Toc166874210)

[eFigure 2. Forest plots of direct comparisons of the risk of AKI following different treatments in lung cancer. 15](#_Toc166874211)

[eFigure 3. Pooled incidence of AKI following targeted treatments in lung cancer based on CTCAE grade. 16](#_Toc166874212)

[eFigure 4. Pooled incidence of increased serum creatinine following regimens containing targeted therapy and chemotherapy alone. 17](#_Toc166874213)

[eFigure 5. Pooled incidence of increased serum creatinine following chemotherapy alone and different categories of targeted therapies. 18](#_Toc166874214)

[eFigure 6. Pooled incidence of increased serum creatinine (CTCAE grade 1-2) following targeted treatments in lung cancer. 19](#_Toc166874215)

[eFigure 7. Pooled incidence of increased serum creatinine (CTCAE grade 3-4) following targeted treatments in lung cancer. 20](#_Toc166874216)

[eFigure 8. Pooled incidence of proteinuria following regimens containing targeted therapy. 21](#_Toc166874217)

[eFigure 9. Forest plots of direct comparisons of proteinuria for all treatments in lung cancer. 22](#_Toc166874218)

[eFigure 10. Pooled incidence of proteinuria following targeted treatments in lung cancer based on CTCAE grade. 23](#_Toc166874219)

[eFigure 11. Pooled incidence of UTI following targeted treatments and chemotherapy alone in lung cancer. 24](#_Toc166874220)

[eFigure 12. Pooled incidence of hypokalemia following targeted treatments in lung cancer. 25](#_Toc166874221)

[eFigure 13. Pooled incidence of hypokalemia following targeted treatments and chemotherapy alone in lung cancer. 26](#_Toc166874222)

[eFigure 14. Pooled incidence of hypokalemia following different categories of targeted treatments and chemotherapy alone in lung cancer. 27](#_Toc166874223)

[eFigure 15. Pooled incidence of hyperkalemia following targeted treatments in lung cancer. 29](#_Toc166874224)

[eFigure 16. Pooled incidence of hyperkalemia following targeted treatments and chemotherapy alone in lung cancer. 30](#_Toc166874225)

[eFigure 17. Pooled incidence of hyponatremia following targeted treatments in lung cancer. 31](#_Toc166874226)

[eFigure 18. Pooled incidence of hyponatremia following targeted treatments and chemotherapy alone in lung cancer. 32](#_Toc166874227)

[eFigure 19. Pooled incidence of hypocalcemia following targeted treatments in lung cancer. 33](#_Toc166874228)

[eFigure 20. Pooled incidence of hypocalcemia following targeted treatments and chemotherapy alone in lung cancer. 34](#_Toc166874229)

[eFigure 21. Pooled incidence of hypercalcemia following targeted treatments in lung cancer. 35](#_Toc166874230)

[eFigure 22. Pooled incidence of hypophosphatemia following targeted treatments in lung cancer. 36](#_Toc166874231)

[eFigure 23. Pooled incidence of hypomagnesemia following targeted treatments in lung cancer. 37](#_Toc166874232)

[eFigure 24. Risk of bias assessment of individual studies using the 7-item Cochrane criteria. 38](#_Toc166874233)

[eFigure 25. Summary graph of the risk of bias assessment of included studies using the Cochrane criteria. 39](#_Toc166874234)

[eFigure 26. Comparison adjusted funnel plot for direct comparisons of AKI following targeted therapies in lung cancer. 40](#_Toc166874235)

[eFigure 27. Funnel plot for increased serum Cr following targeted therapies in lung cancer. 41](#_Toc166874236)

[eFigure 28. Comparison adjusted funnel plot for direct comparisons of proteinuria following targeted therapies in lung cancer. 42](#_Toc166874237)

[eFigure 29. Funnel plot for UTI following targeted therapies in lung cancer. 43](#_Toc166874238)

[eFigure 30. Funnel plot for electrolyte disorders following targeted therapies in lung cancer. 44](#_Toc166874239)

eTable 1. Search terms used in Embase via Ovid.

| 1 | gefitinib.ti,ab,kw |
| --- | --- |
| 2 | erlotinib.ti,ab,kw |
| 3 | osimertinib.ti,ab,kw |
| 4 | AZD9291.ti,ab,kw |
| 5 | necitumumab.ti,ab,kw |
| 6 | afatinib.ti,ab,kw |
| 7 | lorlatinib.ti,ab,kw |
| 8 | brigatinib.ti,ab,kw |
| 9 | crizotinib.ti,ab,kw |
| 10 | dabrafenib.ti,ab,kw |
| 11 | trametinib.ti,ab,kw |
| 12 | nivolumab.ti,ab,kw |
| 13 | pembrolizumab.ti,ab,kw |
| 14 | tislelizumab.ti,ab,kw |
| 15 | sintilimab.ti,ab,kw |
| 16 | furmonertinib.ti,ab,kw |
| 17 | atezolizumab.ti,ab,kw |
| 18 | durvalumab.ti,ab,kw |
| 19 | ramucirumab.ti,ab,kw |
| 20 | bevacizumab.ti,ab,kw |
| 21 | dacomitinib.ti,ab,kw |
| 22 | capmatinib.ti,ab,kw |
| 23 | savolitinib.ti,ab,kw |
| 24 | selpercatinib.ti,ab,kw |
| 25 | pralsetinib.ti,ab,kw |
| 26 | BLU-66.ti,ab,kw |
| 27 | pralsetinib.ti,ab,kw |
| 28 | 1 or 2 or 3 or 4 or 5 or 6 or 7 or 8 or 9 or 10 or 11 or 12 or 13 or 14 or 15 or 16 or 17 or 18 or 19 or 20 or 21 or 22 or 23 or 24 or 25 or 26 or 27 |
| 29 | ‘clinical trial’.ti,ab,kw |
| 30 | ‘random* control* trial’.ti,ab,kw |
| 31 | 29 or 30 |
| 32 | 28 and 31 |
| 33 | safety.mp. |
| 34 | toxicit*.mp. |
| 35 | adverse.mp. |
| 36 | 33 or 34 or 35 |
| 37 | 32 and 36 |
| 38 | lung.mp. |
| 39 | pulmonary.mp. |
| 42 | 38 or 39 |
| 43 | 37 and 40 |

Note: This strategy will be adapted to identify trials in other electronic databases.

eTable 2. General characteristics of studies included in meta-analysis.

| **Author** | **Year** | **Region** | **Registration** | **Population** | **Targeted therapy group** | | | | **Control Group** | | | | **Follow-up** | **Outcomes** |
| --- | --- | --- | --- | --- | --- | --- | --- | --- | --- | --- | --- | --- | --- | --- |
|  |  |  |  |  | **Sample** | **Male** | **Age** | **Regimen** | **Sample** | **Male** | **Age** | **Regimen** |  |  |
| Ahn[1] | 2012 | Korea | NCT00409006 | Advanced NSCLC | 39 | 9 | 56 | pemetrexed plus cisplatin followed by gefitinib | 31 | 6 | 56 | pemetrexed plus cisplatin | 40m | ⑤/⑥ |
| Argiris[2] | 2017 | Greece | NCT00955305 | Advanced non-squamous NSCLC | 75 | 35 | - | CPB plus cixutumumab 6 mg/kg i.v. weekly | 78 | 38 | - | CPB (paclitaxel + carboplatin + bevacizumab) | 48m | ②/③ |
| Ciuleanu[3] | 2018 | Romania | NCT00981058 | Stage IV Squamous NSCLC | 261 | 211 | 62 | gemcitabine and cisplatin with necitumumab | 215 | 191 | 62 | gemcitabine and cisplatin | 39m | ⑪ |
| Ciuleanu[4] | 2013 | Romania | NCT00531960 | Advanced non-squamous NSCLC | 61 | 36 | 58 | bevacizumab plus chemotherapy (emcitabine/cisplatin or carboplatin/paclitaxel) | 63 | 37 | 61 | bevacizumab plus erlotinib | 13m | ② |
| Crinò[5] | 2008 | Italy | NCT00256711 | Advanced NSCLC | 97 | 75 | 74 | gefitinib (250 mg/d orally) | 99 | 73 | 74 | vinorelbine (30 mg/m2 infusion on days 1 and 8 of a 21-day cycle) | 20m | ⑥ |
| Doebele[6] | 2015 | USA | NCT01160744 | Nonsquamous advanced/metastatic NSCLC | 71 | 45 | - | pemetrexed and carboplatin (or cisplatin) plus pemetrexed and carboplatin (or cisplatin) | 69 | 36 | - | ramucirumab (10 mg/kg) plus pemetrexed and carboplatin (or cisplatin) | 20m | ② |
| Du[7] | 2013 | China | - | NSCLC | 36 | 19 | - | bevacizumab (300 mg) with cisplatin (30 mg) | 34 | 19 | - | cisplatin (30 mg) | 10m | ② |
| Ellis[8] | 2014 | Canada | NCT01000025 | Advanced or metastatic NSCLC | 480 | 244 | 63.5 | three previous lines of chemotherapy followed by oral dacomitinib 45 mg once-daily | 240 | 120 | 65.5 | three previous lines of chemotherapy followed by placebo | 32m | ①/④/⑥ |
| Gaafar[9] | 2011 | Egypt | NCT00091156 | Advanced NSCLC | 86 | 67 | 61 | four cycles of platinum-based chemotherapy followed by gefitinib 250 mg/d | 87 | 66 | 62 | four cycles of platinum-based chemotherapy followed by placebo | 60m | ⑤/⑥/⑦/⑨ |
| Garon[10] | 2014 | USA | NCT01168973 | Stage IV NSCLC | 628 | 419 | 62 | docetaxel 75 mg/m² and ramucirumab (10 mg/kg) | 625 | 415 | 61 | docetaxel 75 mg/m² and placebo | 36m | ①/②/④ |
| Goldman[11] | 2020 | USA | NCT02152631 | Stage IV NSCLC | 270 | 163 | 62 | platinum-based chemotherapy followed by 200 mg abemaciclib twice daily | 183 | 109 | 63 | platinum-based chemotherapy followed by 150 mg erlotinib once daily | 33m | ①/④/⑤ |
| Herbst[12] | 2018 | USA | NCT00946712 | Advanced NSCLC | 656 | 385 | 63 | cetuximab (250 mg/m² weekly) plus PCB | 657 | 359 | 63 | PCB (paclitaxel+carboplatin/carboplatin+ bevacizumab) | 60m | ②/⑤/⑥/⑦/⑧/⑪ |
| Johnson[13] | 2013 | USA | NCT00257608 | Advanced NSCLC | 373 | 196 | 64 | four cycles of chemotherapy and bevacizumab plus placebo | 370 | 193 | 64 | four cycles of chemotherapy and bevacizumab plus erlotinib (150 mg per day) | 24m | ①/②/④ |
| Karayama[14] | 2016 | Japan | - | Advanced NSCLC | 55 | 39 | 66 | Pemetrexed 500 mg/m2 | 55 | 35 | 65 | Pemetrexed 500 mg/m2 + bevacizumab 15 mg/kg | 24m | ② |
| Kenmotsu[15] | 2022 | Japan | NCT04181060 | Non-squamous NSCLC | 61 | 23 | 66 | osimertinib (80 mg daily) monotherapy | 61 | 24 | 67 | osimertinib (80 mg daily) plus bevacizumab (15 mg/kg every 3 weeks) | 36m | ②/③/④ |
| Lara[16] | 2016 | USA | - | Advanced NSCLC | 33 | 14 | 74.9 | 150 mg of erlotinib orally daily | 26 | 10 | 70.8 | four cycles of carboplatin and paclitaxel followed by 150 mg of erlotinib orally | 60m | ④ |
| Leighl[17] | 2017 | Canada | - | NSCLC | 44 | 14 | 61.5 | linsitinib 150 mg twice daily plus erlotinib 150 mg once daily | 44 | 12 | 57.5 | placebo plus erlotinib 150 mg once daily | 20m | ④ |
| Lynch[18] | 2009 | USA | - | Advanced NSCLC | 25 | 13 | 64 | erlotinib alone | 25 | 11 | 62 | erlotinib plus bortezomib | 20m | ⑥ |
| Miller[19] | 2012 | USA | NCT00656136 | Advanced metastatic NSCLC | 390 | 159 | 58 | chemotherapy followed by afatinib 50mg/d | 195 | 78 | 59 | chemotherapy followed by placebo | 24m | ⑥ |
| Nakagawa[20] | 2019 | Japa | NCT02411448 | Advanced NSCLC | 224 | 83 | 65 | oral erlotinib (150 mg/day) plus intravenous ramucirumab (10 mg/kg) | 225 | 83 | 64 | oral erlotinib (150 mg/day) plus placebo | 36m | ② |
| Niho[21] | 2012 | Japa | - | Advanced non-squamous NSCLC | 59 | 38 | 60 | carboplatin-paclitaxel | 121 | 77 | 61 | bevacizumab plus carboplatin-paclitaxel | 36m | ⑤ |
| Paz-Ares[22] | 2015 | Spain | NCT00982111 | Stage IV non-squamous NSCLC | 315 | 214 | 61 | 800mg necitumumab was continued after the end of chemotherapy | 318 | 210 | 60 | chemotherapy (pemetrexed +cisplatin) | 36m | ⑪ |
| Paz-Ares[23] | 2017 | Spain | NCT01168973 | Advanced NSCLC | 628 | - | - | ramucirumab (10 mg/kg) plus docetaxel (75 mg/m2) | 625 | - | - | placebo plus docetaxel | 36m | ①/②/④ |
| Pérol[24] | 2012 | France | - | Advanced NSCLC | 154 | 113 | 57.9 | four cycles of cisplatin-gemcitabine followed by gemcitabine 1250 mg/m2 | 155 | 113 | 26.4 | four cycles of cisplatin-gemcitabine followed by daily erlotinib 150 mg/day | 44m | ①/④ |
| Pujol[25] | 2015 | France | NCT00930891 | Extensive small-cell lung cancer | 37 | 26 | 60.1 | chemotherapy alone | 37 | 25 | 61.2 | chemotherapy plus bevacizumab 7.5mg/kg | 30m | ②/④ |
| Ramlau[26] | 2012 | Poland | NCT00532155 | Advanced or metastatic NSCLC | 456 | 305 | 59.6 | aflibercept 6 mg/kg combination with docetaxel 75 mg/m2 | 457 | 300 | 59.6 | placebo combination with docetaxel 75 mg/m2 | 36m | ② |
| Reck[27] | 2016 | Germany | NCT00981058 | Stage IV squamous NSCLC | 545 | 450 | 62 | necitumumab (800 mg) plus gemcitabine-cisplatin | 548 | 458 | 62 | gemcitabine-cisplatin | 32m | ④/⑪ |
| Reck[28] | 2009 | Germany | - | Non-squamous NSCLC | 696 | 223 | 57 | bevacizumab 7.5 mg/kg plus cisplatin/gemcitabine | 347 | 223 | 59 | placebo plus cisplatin/gemcitabine | 18m | ② |
| Sandler[29] | 2006 | USA | NCT00021060 | NSCLC | 417 | 210 | - | bevacizumab plus paclitaxel–carboplatin | 433 | 253 | - | paclitaxel–carboplatin | 42m | ②/⑤ |
| Scagliotti[30] | 2012 | Italy | - | Advanced NSCLC | 480 | 297 | 61 | previously treated with one to two chemotherapies followed by sunitinib 37.5 mg/d plus erlotinib 150 mg/d | 480 | 284 | 61 | previously treated with one to two chemotherapies followed by placebo plus erlotinib 150 mg/d | 30m | ④/⑤/⑥/⑦/⑧/⑨/⑩/⑪ |
| Schuler[31] | 2016 | Germany | NCT01121393 | NSCLC | 186 | 62 | 63 | afatinib 40 mg orally once daily | 97 | 30 | 60.5 | platinum-based chemotherapy | 45m | ⑤/⑥ |
| Sequist[32] | 2011 | USA | NCT00777309 | NSCLC | 84 | 51 | 64 | oral erlotinib (150 mg daily) plus oral tivantinib (360 mg twice daily) | 83 | 49 | 62 | erlotinib plus placebo | 20m | ④ |
| Seto[33] | 2014 | Japan | - | Non-squamous NSCLC | 75 | 30 | 67 | erlotinib 150 mg/day plus bevacizumab 15 mg/kg every 3 weeks | 77 | 26 | 67 | erlotinib 150 mg/day monotherapy | 28m | ② |
| Shaw[34] | 2017 | USA | NCT01828112 | NSCLC | 115 | 47 | 54 | oral ceritinib 750 mg per day fasted (in 21-day treatment cycles) | 116 | 55 | 54 | chemotherapy (intravenous pemetrexed 500 mg/m² or docetaxel 75 mg/m², every 21 days) | 24m | ④ |
| Shi[35] | 2017 | China | NCT01719536 | Advanced EGFR mutation-positive lung adenocarcinoma | 148 | 43 | 56 | oral icotinib | 137 | 42 | 56 | chemotherapy (cisplatin + pemetrexed) | 24m | ⑥ |
| Socinski[36] | 2010 | USA | NR | NSCLC | 30 | 16 | 65 | sunitinib plus BCP | 26 | 14 | 66 | BCP (bevacizumab plus carboplatin plus paclitaxel) | 12m | ⑤ |
| Spigel[37] | 2018 | USA | NR | Advanced NSCLC | 127 | 66 | 66 | erlotinib (150 mg by mouth daily) plus pazopanib (600 mg by mouth daily) | 65 | 38 | 67 | erlotinib (150 mg by mouth daily) plus placebo | 45m | ② |
| Spigel[38] | 2013 | USA | NCT00854308 | Advanced NSCLC | 69 | 40 | 64 | onartuzumab plus erlotinib | 68 | 42 | 63 | placebo plus erlotinib | 18m | ③ |
| Spigel[39] | 2017 | USA | NCT01769391 | Stage IV Squamous NSCLC | 110 | 87 | 66 | necitumumab plus chemotherapy (paclitaxel + carboplatin) | 57 | 44 | 65 | chemotherapy (paclitaxel + carboplatin) | 24m | ⑪ |
| Spigel[40] | 2017 | USA | NCT00609804 | Advanced NSCLC | 24 | 8 | 67 | erlotinib and sorafenib (400 mg orally twice daily) | 28 | 10 | 63 | sorafenib alone | 48m | ③/⑤/⑥ |
| Steendam[41] | 2021 | Netherlands | NCT0277500 | Relapsed non-squamous NSCLC | 22 | 11 | - | docetaxel 75 mg/m2 plus erlotinib 150 mg/day | 23 | 8 | - | docetaxel 75 mg/m2 intravenously on day 1 every 21 days | 12m | ①/④ |
| Stephenson[42] | 2014 | USA | NCT00732810 | NSCLC | 17 | 10 | 63.2 | intravenous (IV) dinaciclib (50 mg/m2) | 33 | 21 | 63.3 | oral erlotinib (150 mg) | 22m | ⑤ |
| Stinchcombe[43] | 2019 | USA | NCT01532089 | Advanced EGFR-Mutant NSCLC | 43 | 12 | 65 | erlotinib plus bevacizumab | 45 | 14 | 63 | erlotinib alone | 60m | ② |
| Tada[44] | 2022 | Japan | UMIN000006252 | Resected Stage II-IIIA NSCLC | 116 | 44 | 64 | gefitinib (250 mg once daily) | 116 | 45 | 64 | cisplatin (80 mg/m2) plus vinorelbine (25 mg/m2) | 108m | ④/⑤/⑥ |
| Takeda[45] | 2010 | Japan | UMINC000000035 | Advanced NSCLC | 298 | 191 | 63 | platinum-doublet chemotherapy | 300 | 192 | 62 | chemotherapy followed by gefitinib 250 mg orally once daily | 60m | ④ |
| Takeda[46] | 2016 | Japan | NCT01351415 | Advanced non-squamous NSCLC | 50 | 33 | 64.5 | bevacizumab plus docetaxel | 50 | 32 | 67 | docetaxel | 30m | ②/④ |
| Thatcher[47] | 2015 | UK | NCT00981058 | Stage IV squamous NSCLC | 545 | 450 | 62 | necitumumab plus chemotherapy (gemcitabine and cisplastin) | 548 | 458 | 62 | chemotherapy (gemcitabine and cisplastin) | 40m | ⑪ |
| von Pawel[48] | 2018 | Germany | NCT01366131 | Non-squamous NSCLC | 52 | - | - | placebo plus bevacizumab (15 mg/kg) and carboplatin/paclitaxel | 52 | - | - | parsatuzumab (600 mg) plus bevacizumab (15 mg/kg) and carboplatin/paclitaxel | 15m | ② |
| Wakelee[49] | 2017 | USA | NCT01496742 | Advanced non-Squamous NSCLC | 69 | 47 | 60 | onartuzumab plus paclitaxel/platinum/bevacizumab | 70 | 34 | 60.5 | placebo plus paclitaxel/platinum/bevacizumab | 16m | ⑤ |
|  |  |  |  |  | 59 | 33 | 66 | onartuzumab plus platinum/pemetrexed | 61 | 26 | 63 | placebo plus platinum/pemetrexed | 16m | ⑤/⑥ |
| Wakelee[50] | 2017 | USA | NCT00324805 | Resected NSCLC | 749 | 375 | 61 | chemotherapy alone | 752 | 371 | 61 | bevacizumab 15 mg/kg plus chemotherapy | 84m | ①/②/③/④/⑤/⑥/⑦/⑧/⑩/⑪ |
| Witta[51] | 2012 | USA | NR | Advanced NSCLC | 65 | 43 | 67 | erlotinib 150mg plus placebo | 67 | 39 | 66 | erlotinib 150 mg plus entinostat 10 mg | 30m | ⑥/⑩ |
| Wu[52] | 2018 | China | NCT01639001 | Advanced NSCLC | 104 | 50 | 48 | orally crizotinib 250 mg twice daily | 103 | 43 | 50 | chemotherapy (pemetrexed plus cisplatin or carboplatin) | 30m | ⑤ |
| Wu[53] | 2015 | China | NCT01342965 | NSCLC | 110 | 42 | 57.5 | oral erlotinib 150 mg once daily | 107 | 42 | 56 | chemotherapy (gemcitabine plus cisplatin) | 36m | ⑥ |
| Yoh[54] | 2016 | Japan | NCT01703091 | Stage IV NSCLC | 76 | 59 | 65.6 | ramucirumab 10 mg/kg followed by docetaxel | 81 | 62 | 64.9 | placebo followed by docetaxel | 30m | ①/②/④ |
| Yoshioka[55] | 2015 | Japan | NCT01377376 | Stage IIIB/IV non-squamous NSCLC | 153 | 102 | 63 | erlotinib plus placebo | 154 | 109 | 63 | erlotinib plus tivantinib | 28m | ⑩ |
| Zhong[56] | 2018 | China | NCT01405079 | Stage II-IIIA (N1-N2) EGFR-mutant NSCLC | 111 | 44 | 58 | geftinib (250 mg once daily) for 24 months | 111 | 45 | 60 | vinorelbine (25 mg/m²) plus cisplatin (75 mg/m²) | 60m | ⑥ |
| Zhou[57] | 2015 | China | NCT01364012 | Advanced or recurrent non-squamous NSCLC | 138 | 75 | 57 | bevacizumab 15 mg/kg plus carboplatin/paclitaxel | 138 | 77 | 56 | placebo plus carboplatin/paclitaxel | 36m | ② |

Outcomes: ①AKI; ②proteinuria; ③urinary tract infection; ④blood creatinine increased; ⑤hyponatraemia; ⑥hypokalaemia; ⑦hyperkalaemia; ⑧hypocalcaemia; ⑨hypercalcaemia; ⑩hypophosphatemia; ⑪hypomagnesaemia.

Abbreviations: NSCLC, non-small cell lung cancer.

eTable 3. Test results of the consistency in the network analysis for acute kidney injury.

| Treatments | Direct Coef. | Std. Err. | Indirect Coef. | Std. Err. | Difference Coef. | Std. Err. | P>\|z\| | tau |
| --- | --- | --- | --- | --- | --- | --- | --- | --- |
| Chemo vs EGFR+chemo | -0.7230 | 0.6543 | -1.5848 | 194.2210 | 0.8618 | 194.2221 | 0.9960 | 0.0000 |
| Chemo vs VEGF+chemo | 0.0109 | 0.2434 | -1.0995 | 489.3731 | 1.1104 | 489.3731 | 0.9980 | 0.0000 |
| EGFR+chemo vs EGFR+chemo | 2.0196 | 1.0491 | 0.2960 | 386.8185 | 1.7236 | 386.8223 | 0.9960 | 0.0000 |
| VEGF+chemo vs VEGF+EGFR+chemo | 1.6122 | 1.5510 | -0.6097 | 989.7853 | 2.2218 | 989.7881 | 0.9980 | 0.0000 |

Note: The targeted therapies were categorized based on the targeted molecules.

Abbreviations: chemo, chemotherapy.

eTable 4. Test results of the consistency in the network analysis for proteinuria.

| Treatments | Direct Coef. | Std. Err. | Indirect Coef. | Std. Err. | Difference Coef. | Std. Err. | P>\|z\| | tau |
| --- | --- | --- | --- | --- | --- | --- | --- | --- |
| Chemo vs VEGF+chemo | 1.4314 | 0.1873 | 1.9280 | 1.5234 | -0.4966 | 1.5372 | 0.7470 | 0.2669 |
| Chemo vs VEGF+EGFR+chemo | 2.3447 | 1.4834 | 1.8448 | 0.4075 | 0.4999 | 1.5384 | 0.7450 | 0.2668 |
| EGFR vs VEGF+EGFR | 1.8650 | 0.3032 | 4.6712 | 87.9225 | -2.8062 | 87.9224 | 0.9750 | 0.2619 |
| VEGF+chemo vs VEGF+EGFR | 0.6931 | 0.9233 | -0.7597 | 43.4572 | 1.4528 | 43.4671 | 0.9730 | 0.2619 |
| VEGF+chemo vs VEGF+EGFR+chemo | 0.4134 | 0.3520 | 0.9130 | 1.4951 | -0.4996 | 1.5383 | 0.7450 | 0.2668 |

Note: The targeted therapies were categorized based on the targeted molecules.

Abbreviations: chemo, chemotherapy.

eFigure 1. Pooled incidence of AKI following regimens containing targeted therapy and chemotherapy alone.


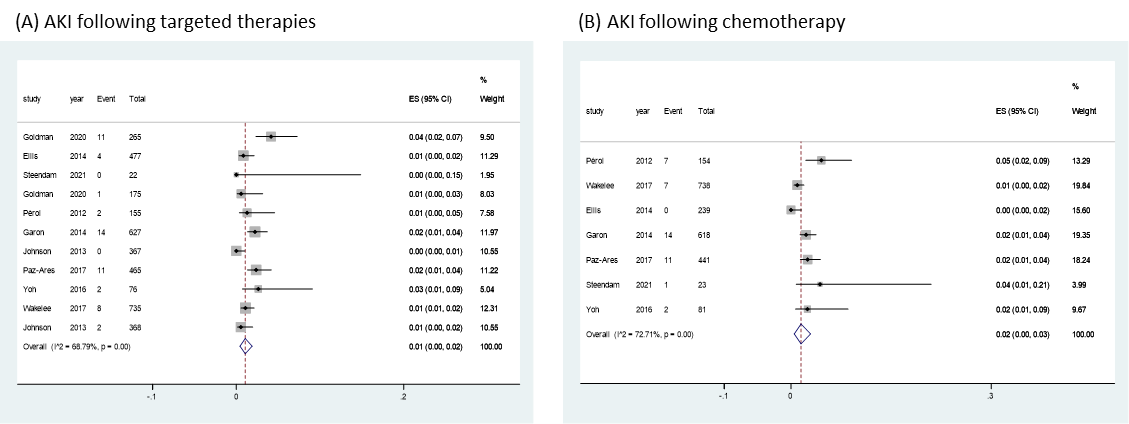


Note: The pooled incidence of AKI following targeted therapy (A) and chemotherapy alone (B) form the included studies that had reported the occurrence of AKI.

eFigure 2. Forest plots of direct comparisons of the risk of AKI following different treatments in lung cancer.


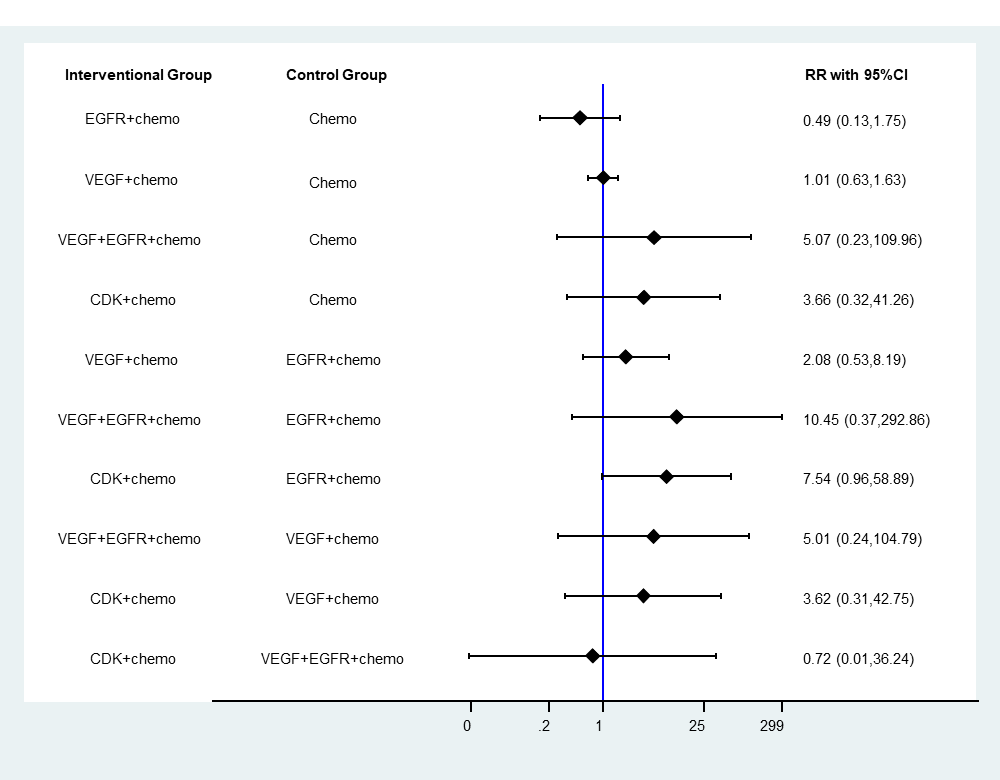


Note: The targeted therapies were categorized based on the targeted molecules.

Abbreviations: CDK, cyclin dependent kinase; chemo, chemotherapy; EGFR, epidermal growth factor receptor; VEGF, vascular endothelial growth factor.

eFigure 3. Pooled incidence of AKI following targeted treatments in lung cancer based on CTCAE grade.


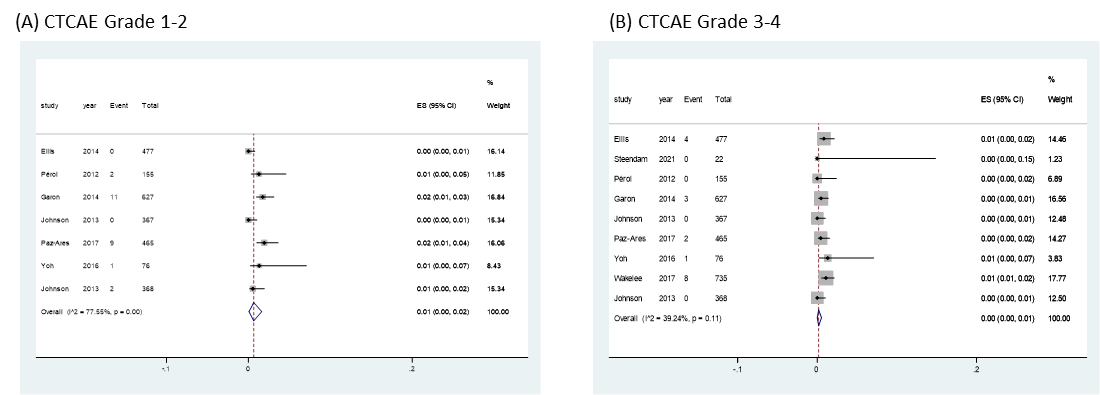


eFigure 4. Pooled incidence of increased serum creatinine following regimens containing targeted therapy and chemotherapy alone.

Note: The targeted therapies were categorized based on the targeted molecules.

Abbreviations: chemo, chemotherapy.

eFigure 5. Pooled incidence of increased serum creatinine following chemotherapy alone and different categories of targeted therapies.

Note: The targeted therapies were categorized based on the targeted molecules.

Abbreviations: CDK, cyclin dependent kinase; chemo, chemotherapy; EGFR, epidermal growth factor receptor; IGF-R, insulin-like growth factor receptor; VEGF, vascular endothelial growth factor.

eFigure 6. Pooled incidence of increased serum creatinine (CTCAE grade 1-2) following targeted treatments in lung cancer.

Note: The targeted therapies were categorized based on the targeted molecules.

eFigure 7. Pooled incidence of increased serum creatinine (CTCAE grade 3-4) following targeted treatments in lung cancer.

Note: The targeted therapies were categorized based on the targeted molecules.

eFigure 8. Pooled incidence of proteinuria following regimens containing targeted therapy.

eFigure 9. Forest plots of direct comparisons of proteinuria for all treatments in lung cancer.


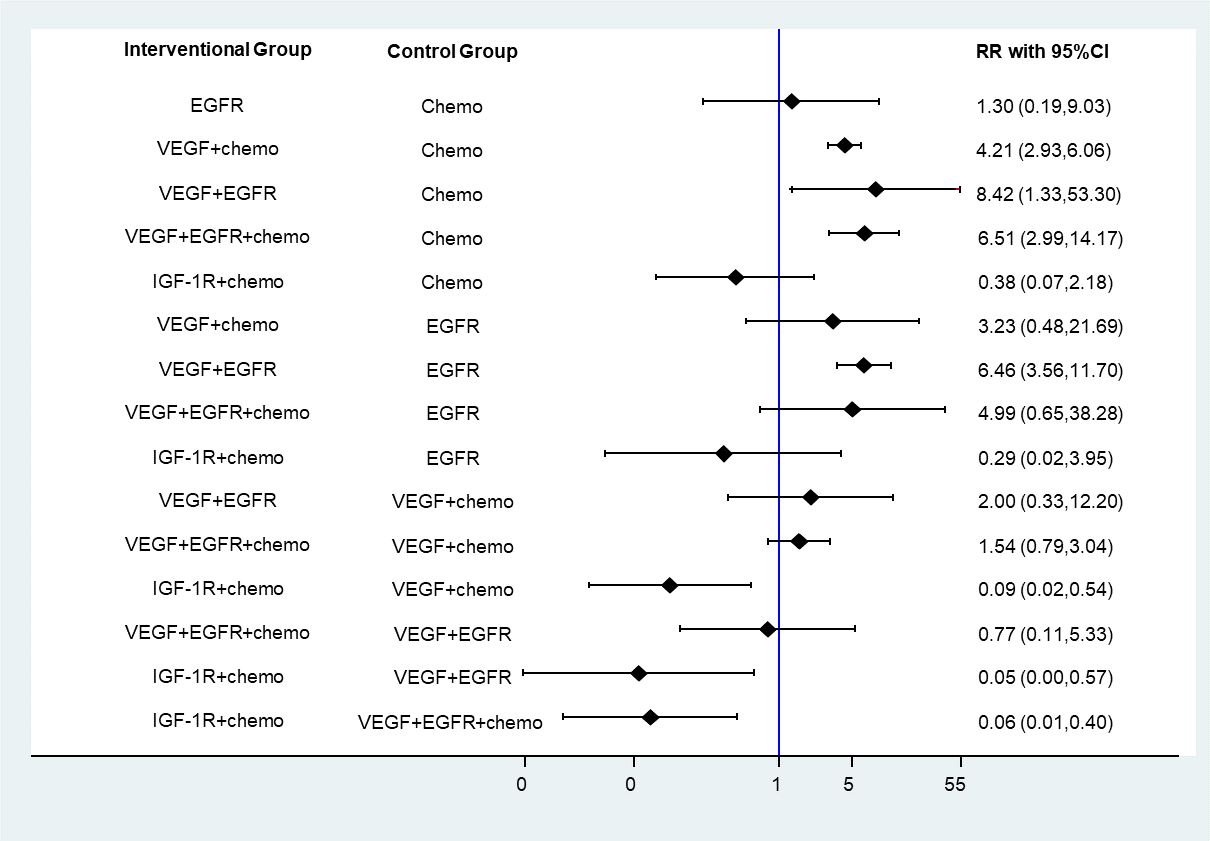


Note: The targeted therapies were categorized based on the targeted molecules.

Abbreviations: CDK, cyclin dependent kinase; chemo, chemotherapy; EGFR, epidermal growth factor receptor; IGF-R, insulin-like growth factor receptor; VEGF, vascular endothelial growth factor.

eFigure 10. Pooled incidence of proteinuria following targeted treatments in lung cancer based on CTCAE grade.


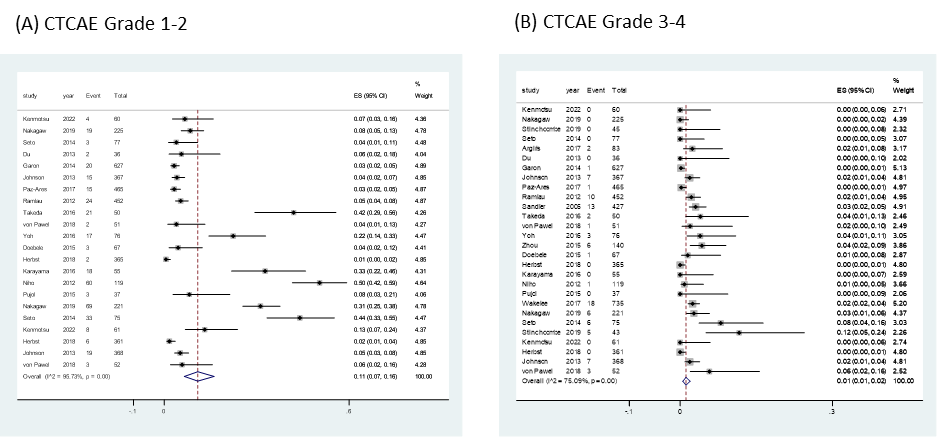


eFigure 11. Pooled incidence of UTI following targeted treatments and chemotherapy alone in lung cancer.


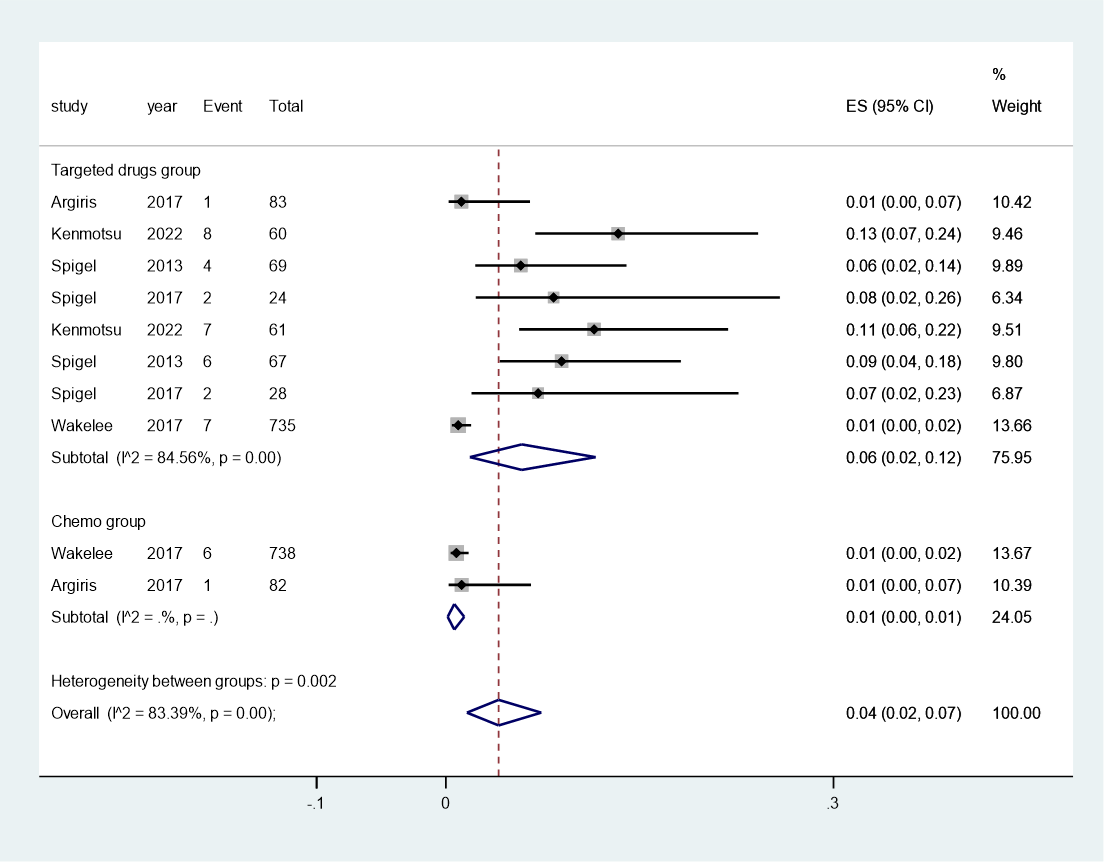


eFigure 12. Pooled incidence of hypokalemia following targeted treatments in lung cancer.


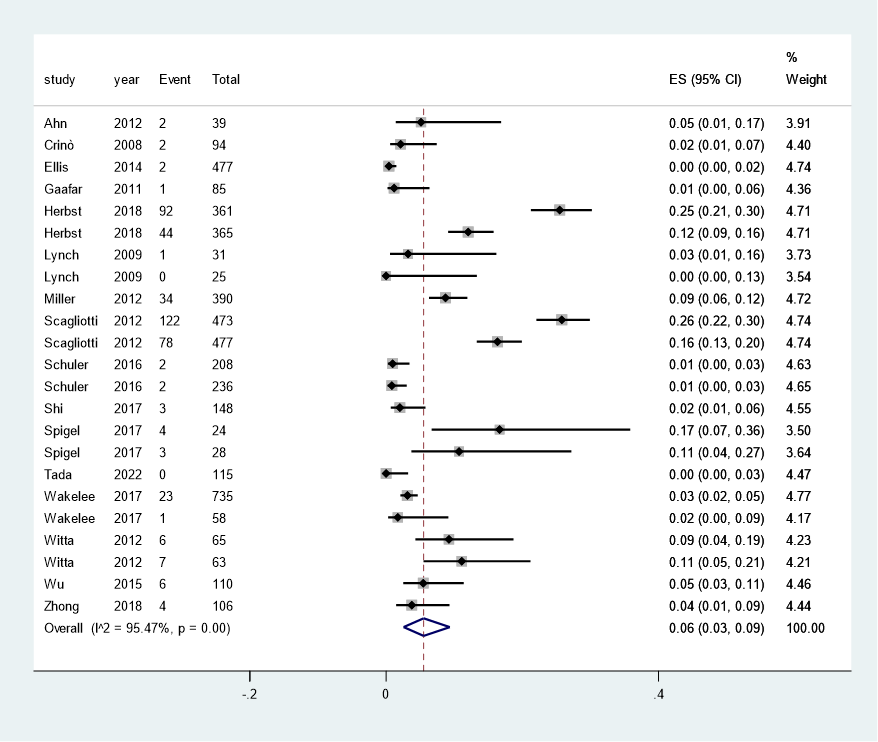


eFigure 13. Pooled incidence of hypokalemia following targeted treatments and chemotherapy alone in lung cancer.


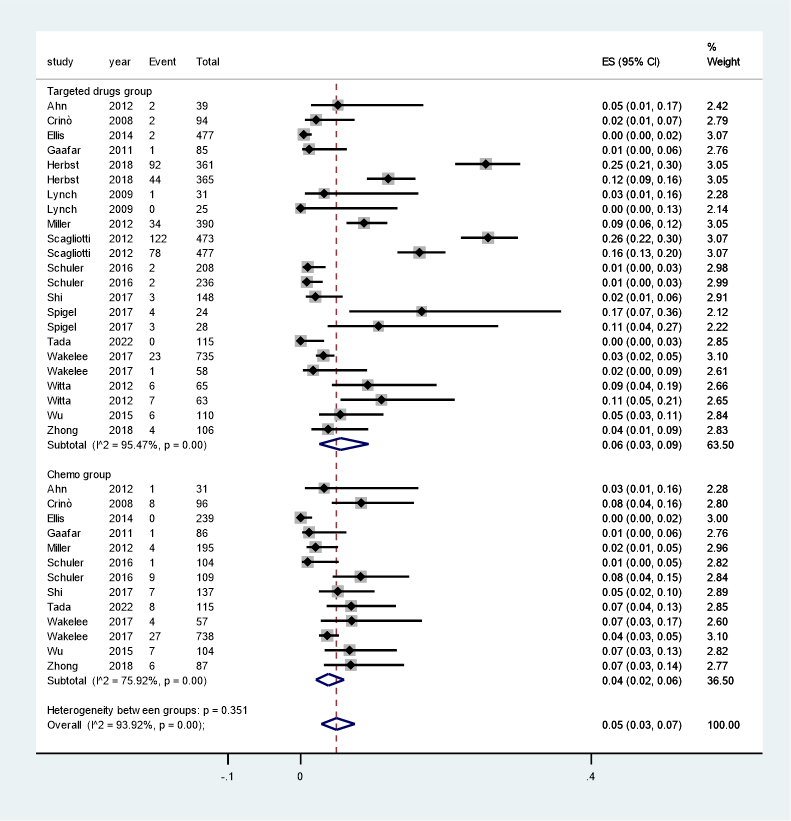


eFigure 14. Pooled incidence of hypokalemia following different categories of targeted treatments and chemotherapy alone in lung cancer.


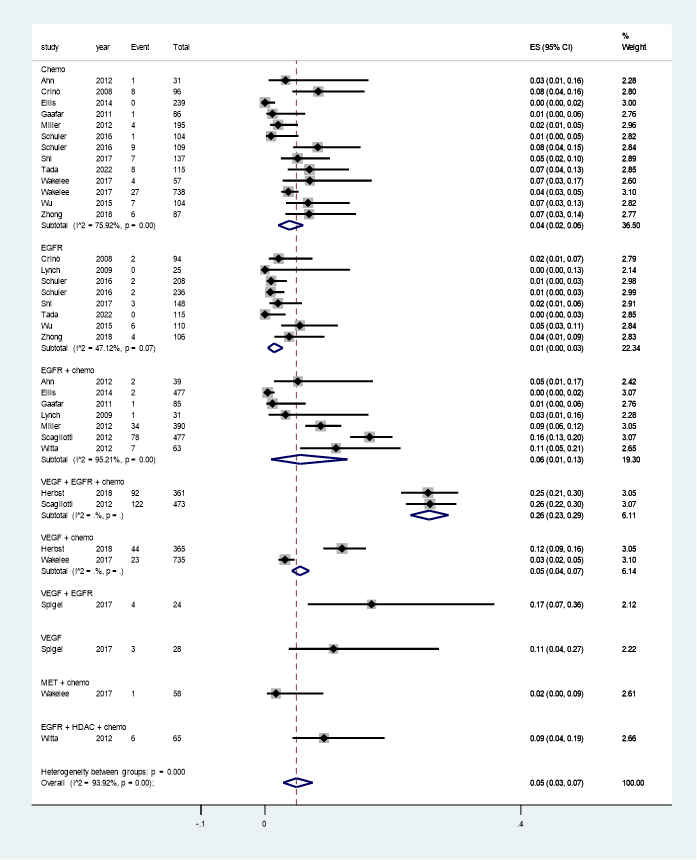


Abbreviations: CDK, cyclin dependent kinase; chemo, chemotherapy; EGFR, epidermal growth factor receptor; HDAC, histone deacetylase; Met, hepatocyte growth factor receptor; VEGF, vascular endothelial growth factor.

eFigure 15. Pooled incidence of hyperkalemia following targeted treatments in lung cancer.


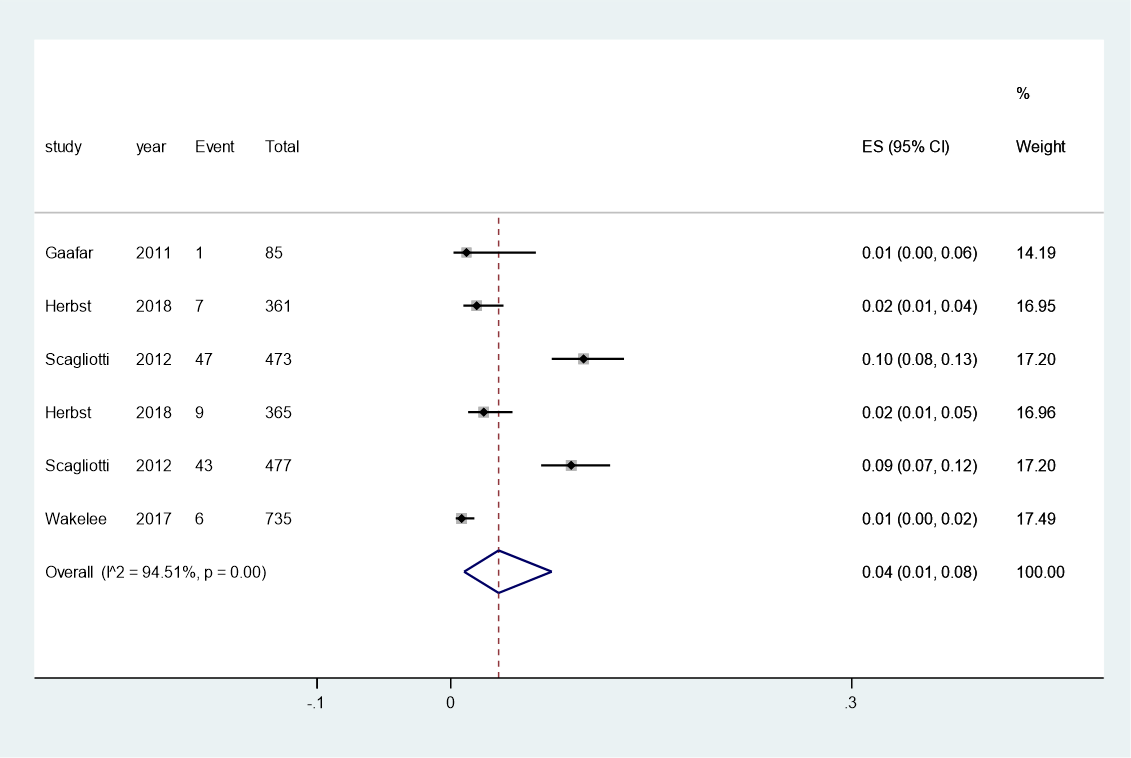


eFigure 16. Pooled incidence of hyperkalemia following targeted treatments and chemotherapy alone in lung cancer.


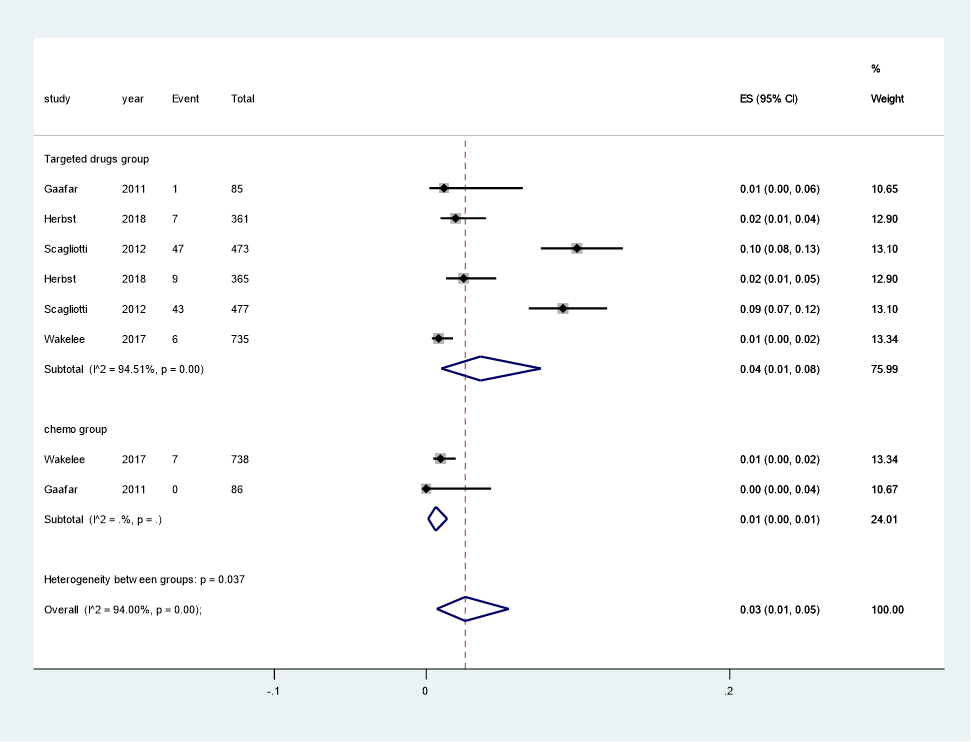


eFigure 17. Pooled incidence of hyponatremia following targeted treatments in lung cancer.


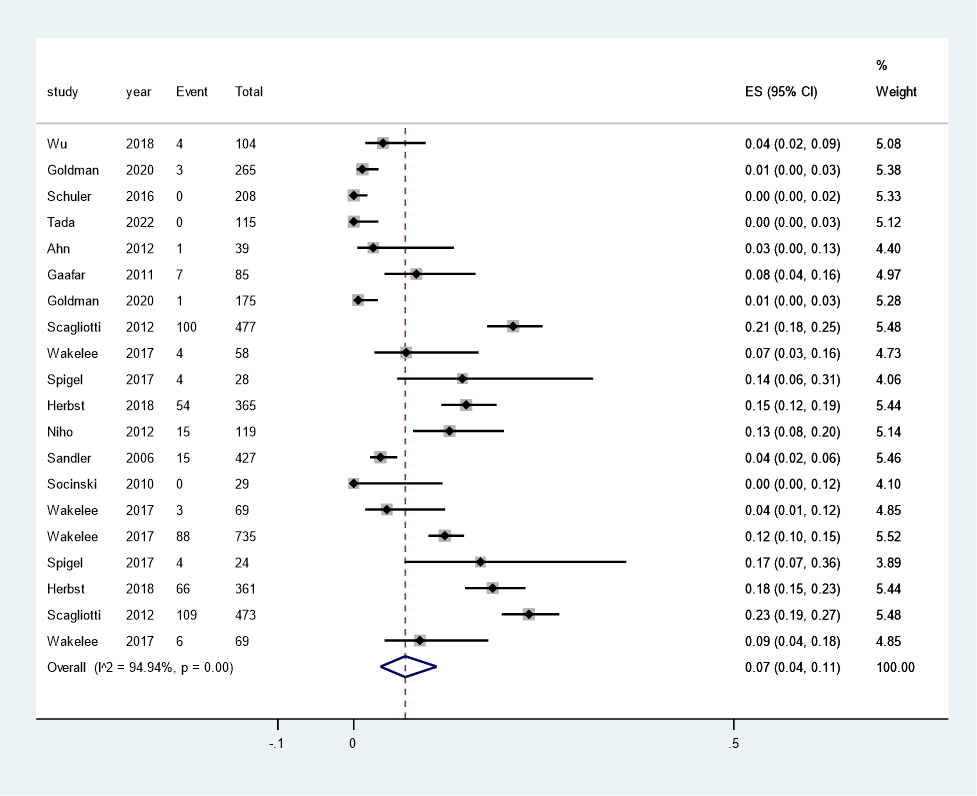


eFigure 18. Pooled incidence of hyponatremia following targeted treatments and chemotherapy alone in lung cancer.


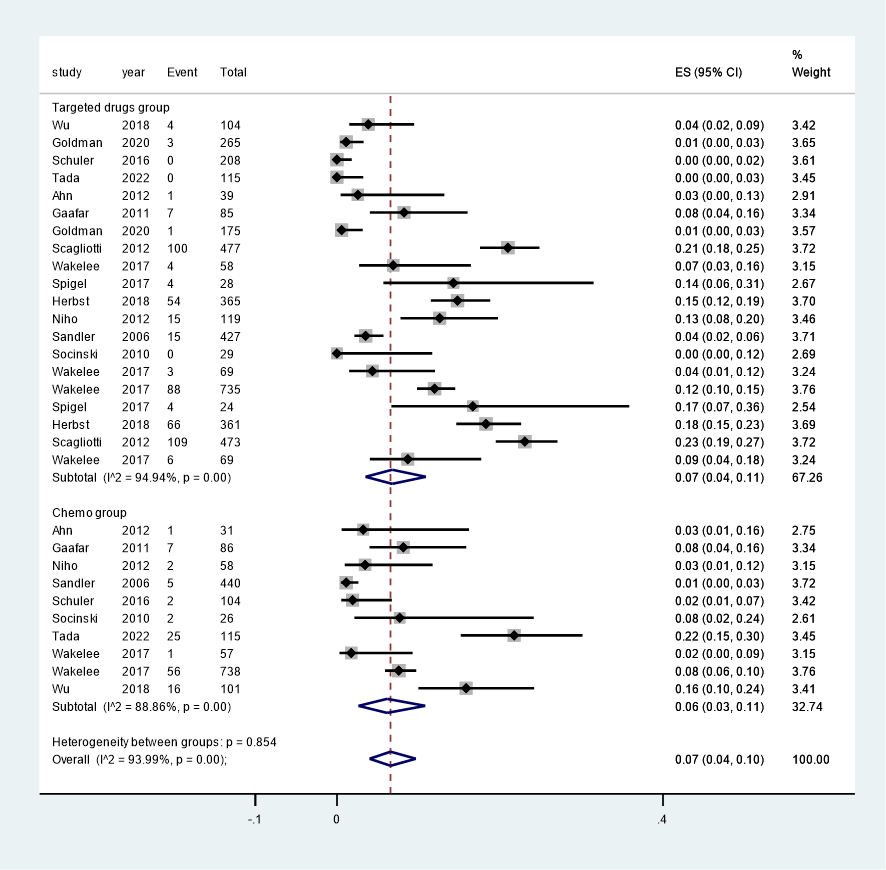


eFigure 19. Pooled incidence of hypocalcemia following targeted treatments in lung cancer.


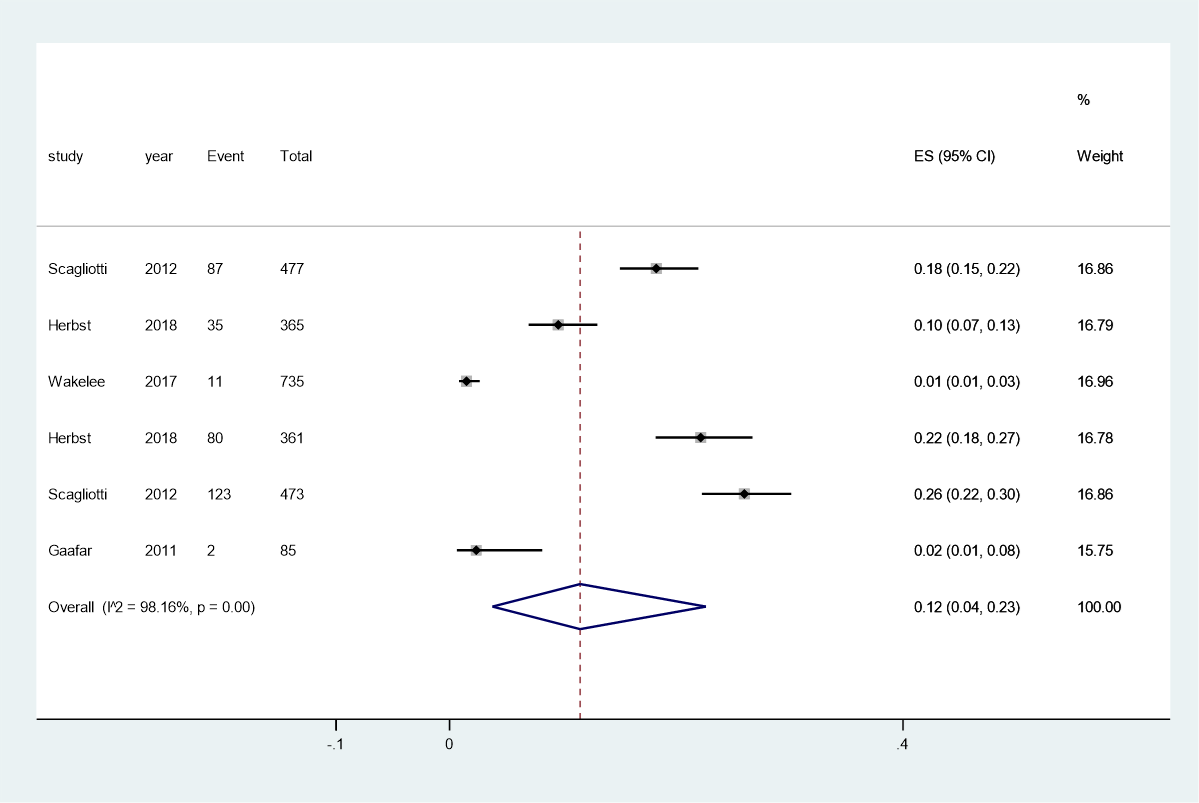


eFigure 20. Pooled incidence of hypocalcemia following targeted treatments and chemotherapy alone in lung cancer.


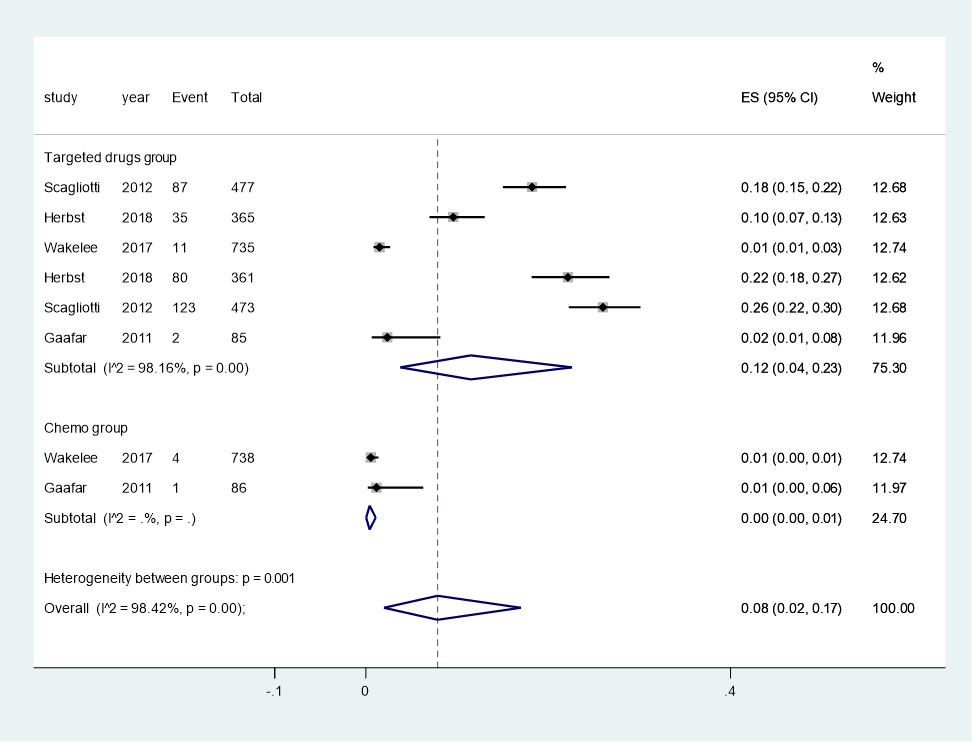


eFigure 21. Pooled incidence of hypercalcemia following targeted treatments in lung cancer.


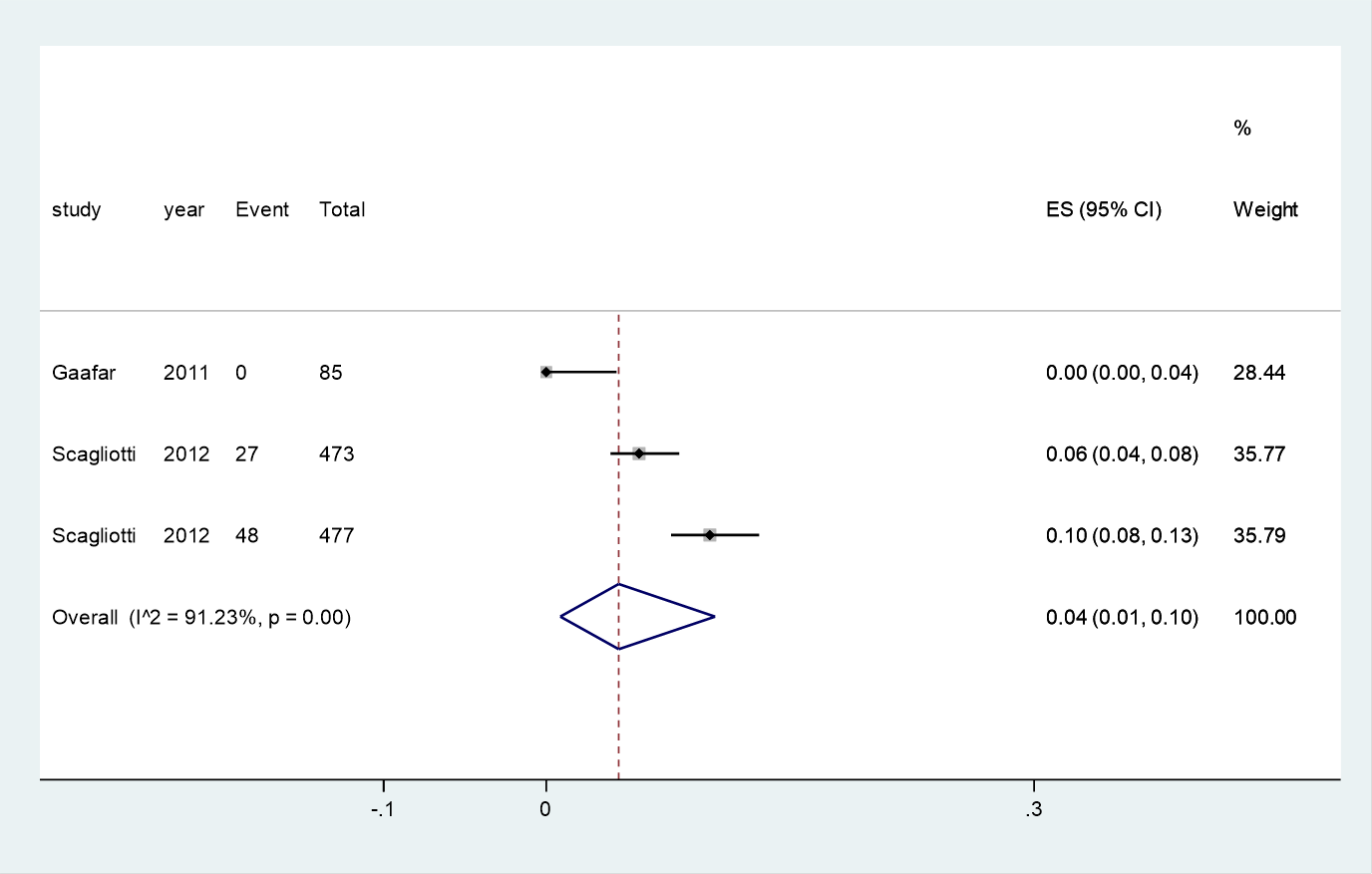


eFigure 22. Pooled incidence of hypophosphatemia following targeted treatments in lung cancer.


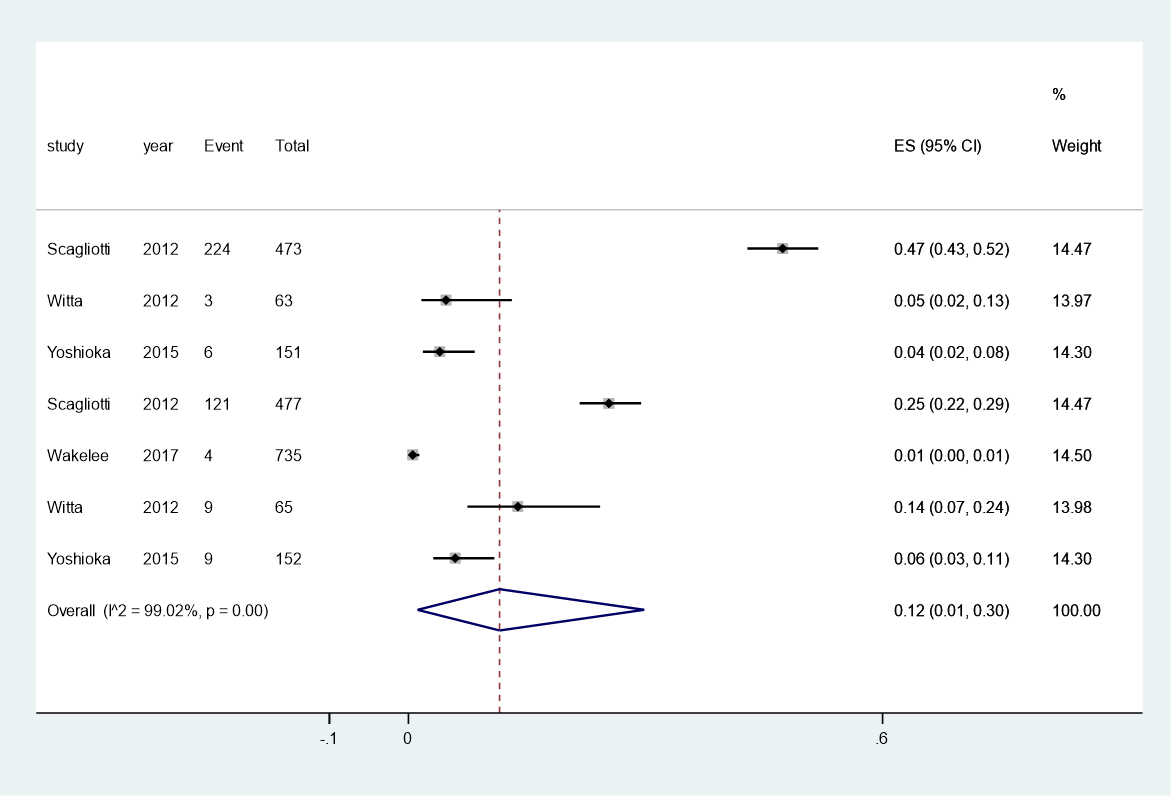


eFigure 23. Pooled incidence of hypomagnesemia following targeted treatments in lung cancer.


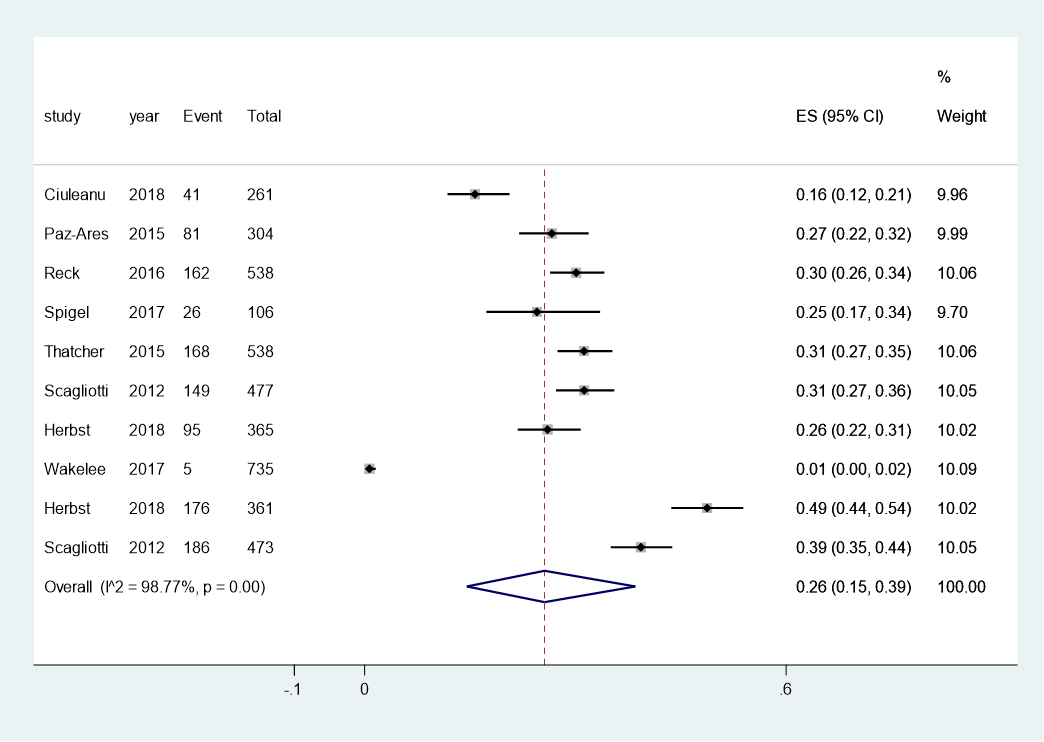


eFigure 24. Risk of bias assessment of individual studies using the 7-item Cochrane criteria.


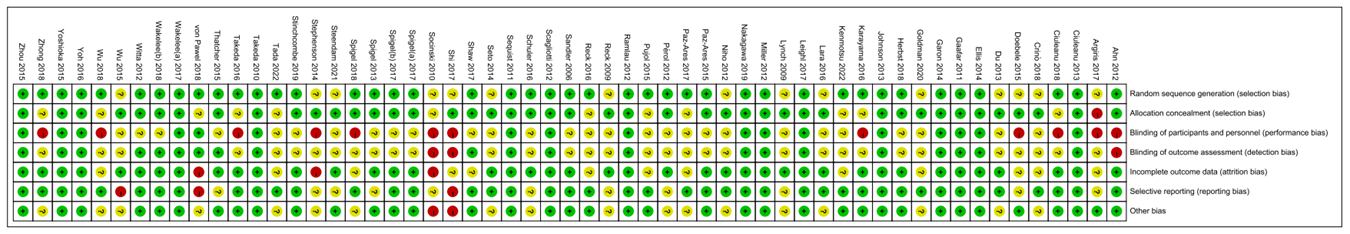


eFigure 25. Summary graph of the risk of bias assessment of included studies using the Cochrane criteria.


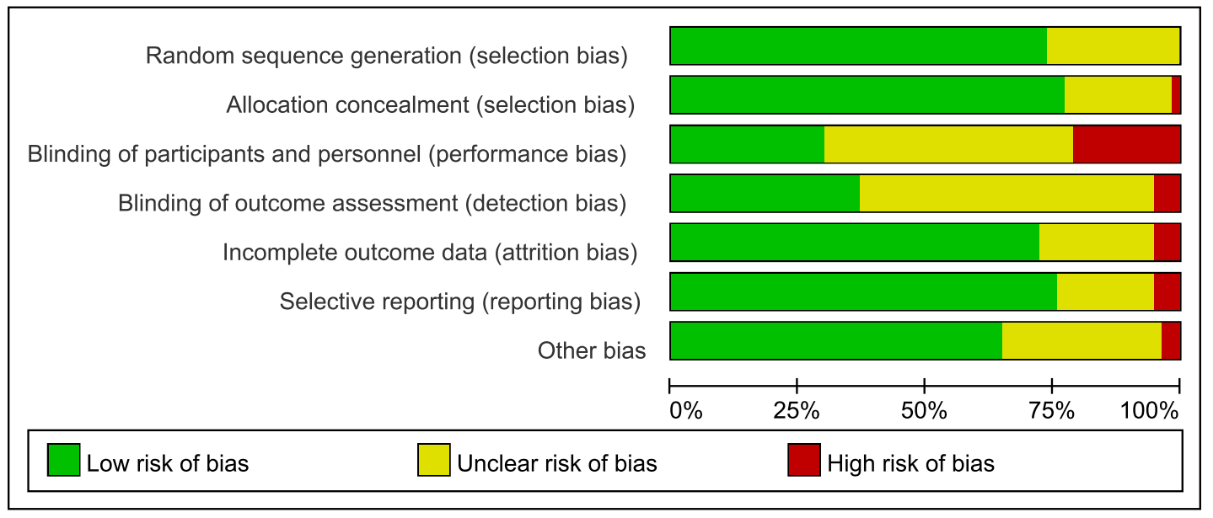


eFigure 26. Comparison adjusted funnel plot for direct comparisons of AKI following targeted therapies in lung cancer.

Note: The treatments are as follows: A: Chemo; B: EGFR + chemo; C: VEGF + chemo; D: VEGF + EGFR + chemo; E: CDK + chemo.

Abbreviations: CDK, cyclin dependent kinase; chemo, chemotherapy; EGFR, epidermal growth factor receptor; VEGF, vascular endothelial growth factor.

eFigure 27. Funnel plot for increased serum Cr following targeted therapies in lung cancer.

eFigure 28. Comparison adjusted funnel plot for direct comparisons of proteinuria following targeted therapies in lung cancer.

Note: The treatments are as follows: A: chemo; B: EGFR; C: VEGF+chemo; D: VEGF+EGFR; E: VEGF+EGFR+chemo; F: IGF-1R+chemo.

Abbreviations: CDK, cyclin dependent kinase; chemo, chemotherapy; EGFR, epidermal growth factor receptor; IGF-R, insulin-like growth factor receptor; VEGF, vascular endothelial growth factor.

eFigure 29. Funnel plot for UTI following targeted therapies in lung cancer.

eFigure 30. Funnel plot for electrolyte disorders following targeted therapies in lung cancer.

**References**

1. Ahn, M.J., et al., *Randomized phase II trial of first-line treatment with pemetrexed-cisplatin, followed sequentially by gefitinib or pemetrexed, in East Asian, never-smoker patients with advanced non-small cell lung cancer.* Lung Cancer, 2012. **77**(2): p. 346-52.

2. Argiris, A., et al., *Phase II randomized trial of carboplatin, paclitaxel, bevacizumab with or without cixutumumab (IMC-A12) in patients with advanced non-squamous, non-small-cell lung cancer: a trial of the ECOG-ACRIN Cancer Research Group (E3508).* Ann Oncol, 2017. **28**(12): p. 3037-3043.

3. Ciuleanu, T., et al., *Efficacy and Safety of Necitumumab Continuation Therapy in the Phase III SQUIRE Study of Patients With Stage IV Squamous Non-Small-Cell Lung Cancer.* Clin Lung Cancer, 2018. **19**(2): p. 130-138.e2.

4. Ciuleanu, T., et al., *A phase II study of erlotinib in combination with bevacizumab versus chemotherapy plus bevacizumab in the first-line treatment of advanced non-squamous non-small cell lung cancer.* Lung Cancer, 2013. **82**(2): p. 276-81.

5. Crinò, L., et al., *Gefitinib versus vinorelbine in chemotherapy-naive elderly patients with advanced non-small-cell lung cancer (INVITE): a randomized, phase II study.* J Clin Oncol, 2008. **26**(26): p. 4253-60.

6. Doebele, R.C., et al., *Phase 2, randomized, open-label study of ramucirumab in combination with first-line pemetrexed and platinum chemotherapy in patients with nonsquamous, advanced/metastatic non-small cell lung cancer.* Cancer, 2015. **121**(6): p. 883-92.

7. Du, N., et al., *Intrapleural combination therapy with bevacizumab and cisplatin for non-small cell lung cancer‑mediated malignant pleural effusion.* Oncol Rep, 2013. **29**(6): p. 2332-40.

8. Ellis, P.M., et al., *Dacomitinib compared with placebo in pretreated patients with advanced or metastatic non-small-cell lung cancer (NCIC CTG BR.26): a double-blind, randomised, phase 3 trial.* Lancet Oncol, 2014. **15**(12): p. 1379-88.

9. Gaafar, R.M., et al., *A double-blind, randomised, placebo-controlled phase III intergroup study of gefitinib in patients with advanced NSCLC, non-progressing after first line platinum-based chemotherapy (EORTC 08021/ILCP 01/03).* Eur J Cancer, 2011. **47**(15): p. 2331-40.

10. Garon, E.B., et al., *Ramucirumab plus docetaxel versus placebo plus docetaxel for second-line treatment of stage IV non-small-cell lung cancer after disease progression on platinum-based therapy (REVEL): a multicentre, double-blind, randomised phase 3 trial.* Lancet, 2014. **384**(9944): p. 665-73.

11. Goldman, J.W., et al., *A Randomized Phase III Study of Abemaciclib Versus Erlotinib in Patients with Stage IV Non-small Cell Lung Cancer With a Detectable KRAS Mutation Who Failed Prior Platinum-Based Therapy: JUNIPER.* Front Oncol, 2020. **10**: p. 578756.

12. Herbst, R.S., et al., *Cetuximab plus carboplatin and paclitaxel with or without bevacizumab versus carboplatin and paclitaxel with or without bevacizumab in advanced NSCLC (SWOG S0819): a randomised, phase 3 study.* Lancet Oncol, 2018. **19**(1): p. 101-114.

13. Johnson, B.E., et al., *ATLAS: randomized, double-blind, placebo-controlled, phase IIIB trial comparing bevacizumab therapy with or without erlotinib, after completion of chemotherapy, with bevacizumab for first-line treatment of advanced non-small-cell lung cancer.* J Clin Oncol, 2013. **31**(31): p. 3926-34.

14. Karayama, M., et al., *Maintenance therapy with pemetrexed and bevacizumab versus pemetrexed monotherapy after induction therapy with carboplatin, pemetrexed, and bevacizumab in patients with advanced non-squamous non small cell lung cancer.* Eur J Cancer, 2016. **58**: p. 30-7.

15. Kenmotsu, H., et al., *Randomized Phase 2 Study of Osimertinib Plus Bevacizumab Versus Osimertinib for Untreated Patients With Nonsquamous NSCLC Harboring EGFR Mutations: WJOG9717L Study.* J Thorac Oncol, 2022. **17**(9): p. 1098-1108.

16. Lara, P.N., Jr., et al., *SWOG S0709: Randomized Phase II Trial of Erlotinib versus Erlotinib Plus Carboplatin/Paclitaxel in Patients with Advanced Non-Small Cell Lung Cancer and Impaired Performance Status as Selected by a Serum Proteomics Assay.* J Thorac Oncol, 2016. **11**(3): p. 420-5.

17. Leighl, N.B., et al., *Phase 2 Study of Erlotinib in Combination With Linsitinib (OSI-906) or Placebo in Chemotherapy-Naive Patients With Non-Small-Cell Lung Cancer and Activating Epidermal Growth Factor Receptor Mutations.* Clin Lung Cancer, 2017. **18**(1): p. 34-42.e2.

18. Lynch, T.J., et al., *A randomized phase 2 study of erlotinib alone and in combination with bortezomib in previously treated advanced non-small cell lung cancer.* J Thorac Oncol, 2009. **4**(8): p. 1002-9.

19. Miller, V.A., et al., *Afatinib versus placebo for patients with advanced, metastatic non-small-cell lung cancer after failure of erlotinib, gefitinib, or both, and one or two lines of chemotherapy (LUX-Lung 1): a phase 2b/3 randomised trial.* Lancet Oncol, 2012. **13**(5): p. 528-38.

20. Nakagawa, K., et al., *Ramucirumab plus erlotinib in patients with untreated, EGFR-mutated, advanced non-small-cell lung cancer (RELAY): a randomised, double-blind, placebo-controlled, phase 3 trial.* Lancet Oncol, 2019. **20**(12): p. 1655-1669.

21. Niho, S., et al., *Randomized phase II study of first-line carboplatin-paclitaxel with or without bevacizumab in Japanese patients with advanced non-squamous non-small-cell lung cancer.* Lung Cancer, 2012. **76**(3): p. 362-7.

22. Paz-Ares, L., et al., *Necitumumab plus pemetrexed and cisplatin as first-line therapy in patients with stage IV non-squamous non-small-cell lung cancer (INSPIRE): an open-label, randomised, controlled phase 3 study.* Lancet Oncol, 2015. **16**(3): p. 328-37.

23. Paz-Ares, L.G., et al., *Treatment outcomes by histology in REVEL: A randomized phase III trial of Ramucirumab plus docetaxel for advanced non-small cell lung cancer.* Lung Cancer, 2017. **112**: p. 126-133.

24. Pérol, M., et al., *Randomized, phase III study of gemcitabine or erlotinib maintenance therapy versus observation, with predefined second-line treatment, after cisplatin-gemcitabine induction chemotherapy in advanced non-small-cell lung cancer.* J Clin Oncol, 2012. **30**(28): p. 3516-24.

25. Pujol, J.L., et al., *Randomized phase II-III study of bevacizumab in combination with chemotherapy in previously untreated extensive small-cell lung cancer: results from the IFCT-0802 trial†.* Ann Oncol, 2015. **26**(5): p. 908-914.

26. Ramlau, R., et al., *Aflibercept and Docetaxel versus Docetaxel alone after platinum failure in patients with advanced or metastatic non-small-cell lung cancer: a randomized, controlled phase III trial.* J Clin Oncol, 2012. **30**(29): p. 3640-7.

27. Reck, M., et al., *The Effect of Necitumumab in Combination with Gemcitabine plus Cisplatin on Tolerability and on Quality of Life: Results from the Phase 3 SQUIRE Trial.* J Thorac Oncol, 2016. **11**(6): p. 808-18.

28. Reck, M., et al., *Phase III trial of cisplatin plus gemcitabine with either placebo or bevacizumab as first-line therapy for nonsquamous non-small-cell lung cancer: AVAil.* J Clin Oncol, 2009. **27**(8): p. 1227-34.

29. Sandler, A., et al., *Paclitaxel-carboplatin alone or with bevacizumab for non-small-cell lung cancer.* N Engl J Med, 2006. **355**(24): p. 2542-50.

30. Scagliotti, G.V., et al., *Sunitinib plus erlotinib versus placebo plus erlotinib in patients with previously treated advanced non-small-cell lung cancer: a phase III trial.* J Clin Oncol, 2012. **30**(17): p. 2070-8.

31. Schuler, M., et al., *First-Line Afatinib versus Chemotherapy in Patients with Non-Small Cell Lung Cancer and Common Epidermal Growth Factor Receptor Gene Mutations and Brain Metastases.* J Thorac Oncol, 2016. **11**(3): p. 380-90.

32. Sequist, L.V., et al., *Randomized phase II study of erlotinib plus tivantinib versus erlotinib plus placebo in previously treated non-small-cell lung cancer.* J Clin Oncol, 2011. **29**(24): p. 3307-15.

33. Seto, T., et al., *Erlotinib alone or with bevacizumab as first-line therapy in patients with advanced non-squamous non-small-cell lung cancer harbouring EGFR mutations (JO25567): an open-label, randomised, multicentre, phase 2 study.* Lancet Oncol, 2014. **15**(11): p. 1236-44.

34. Shaw, A.T., et al., *Ceritinib versus chemotherapy in patients with ALK-rearranged non-small-cell lung cancer previously given chemotherapy and crizotinib (ASCEND-5): a randomised, controlled, open-label, phase 3 trial.* Lancet Oncol, 2017. **18**(7): p. 874-886.

35. Shi, Y.K., et al., *First-line icotinib versus cisplatin/pemetrexed plus pemetrexed maintenance therapy for patients with advanced EGFR mutation-positive lung adenocarcinoma (CONVINCE): a phase 3, open-label, randomized study.* Ann Oncol, 2017. **28**(10): p. 2443-2450.

36. Socinski, M.A., et al., *Safety and efficacy of combining sunitinib with bevacizumab + paclitaxel/carboplatin in non-small cell lung cancer.* J Thorac Oncol, 2010. **5**(3): p. 354-60.

37. Spigel, D.R., et al., *Erlotinib plus either pazopanib or placebo in patients with previously treated advanced non-small cell lung cancer: A randomized, placebo-controlled phase 2 trial with correlated serum proteomic signatures.* Cancer, 2018. **124**(11): p. 2355-2364.

38. Spigel, D.R., et al., *Randomized phase II trial of Onartuzumab in combination with erlotinib in patients with advanced non-small-cell lung cancer.* J Clin Oncol, 2013. **31**(32): p. 4105-14.

39. Spigel, D.R., et al., *An Open-Label, Randomized, Controlled Phase II Study of Paclitaxel-Carboplatin Chemotherapy With Necitumumab Versus Paclitaxel-Carboplatin Alone in First-Line Treatment of Patients With Stage IV Squamous Non-Small-Cell Lung Cancer.* Clin Lung Cancer, 2017. **18**(5): p. 480-488.

40. Spigel, D.R., et al., *Sorafenib and continued erlotinib or sorafenib alone in patients with advanced non-small cell lung cancer progressing on erlotinib: A randomized phase II study of the Sarah Cannon Research Institute (SCRI).* Lung Cancer, 2017. **113**: p. 79-84.

41. Steendam, C.M.J., et al., *Randomized phase III study of docetaxel versus docetaxel plus intercalated erlotinib in patients with relapsed non-squamous non-small cell lung carcinoma.* Lung Cancer, 2021. **160**: p. 44-49.

42. Stephenson, J.J., et al., *Randomized phase 2 study of the cyclin-dependent kinase inhibitor dinaciclib (MK-7965) versus erlotinib in patients with non-small cell lung cancer.* Lung Cancer, 2014. **83**(2): p. 219-23.

43. Stinchcombe, T.E., et al., *Effect of Erlotinib Plus Bevacizumab vs Erlotinib Alone on Progression-Free Survival in Patients With Advanced EGFR-Mutant Non-Small Cell Lung Cancer: A Phase 2 Randomized Clinical Trial.* JAMA Oncol, 2019. **5**(10): p. 1448-1455.

44. Tada, H., et al., *Randomized Phase III Study of Gefitinib Versus Cisplatin Plus Vinorelbine for Patients With Resected Stage II-IIIA Non-Small-Cell Lung Cancer With EGFR Mutation (IMPACT).* J Clin Oncol, 2022. **40**(3): p. 231-241.

45. Takeda, K., et al., *Randomized phase III trial of platinum-doublet chemotherapy followed by gefitinib compared with continued platinum-doublet chemotherapy in Japanese patients with advanced non-small-cell lung cancer: results of a west Japan thoracic oncology group trial (WJTOG0203).* J Clin Oncol, 2010. **28**(5): p. 753-60.

46. Takeda, M., et al., *Bevacizumab beyond disease progression after first-line treatment with bevacizumab plus chemotherapy in advanced nonsquamous non-small cell lung cancer (West Japan Oncology Group 5910L): An open-label, randomized, phase 2 trial.* Cancer, 2016. **122**(7): p. 1050-9.

47. Thatcher, N., et al., *Necitumumab plus gemcitabine and cisplatin versus gemcitabine and cisplatin alone as first-line therapy in patients with stage IV squamous non-small-cell lung cancer (SQUIRE): an open-label, randomised, controlled phase 3 trial.* Lancet Oncol, 2015. **16**(7): p. 763-74.

48. von Pawel, J., et al., *Randomized Phase II Trial of Parsatuzumab (Anti-EGFL7) or Placebo in Combination with Carboplatin, Paclitaxel, and Bevacizumab for First-Line Nonsquamous Non-Small Cell Lung Cancer.* Oncologist, 2018. **23**(6): p. 654-e58.

49. Wakelee, H., et al., *Efficacy and Safety of Onartuzumab in Combination With First-Line Bevacizumab- or Pemetrexed-Based Chemotherapy Regimens in Advanced Non-Squamous Non-Small-Cell Lung Cancer.* Clin Lung Cancer, 2017. **18**(1): p. 50-59.

50. Wakelee, H.A., et al., *Adjuvant chemotherapy with or without bevacizumab in patients with resected non-small-cell lung cancer (E1505): an open-label, multicentre, randomised, phase 3 trial.* Lancet Oncol, 2017. **18**(12): p. 1610-1623.

51. Witta, S.E., et al., *Randomized phase II trial of erlotinib with and without entinostat in patients with advanced non-small-cell lung cancer who progressed on prior chemotherapy.* J Clin Oncol, 2012. **30**(18): p. 2248-55.

52. Wu, Y.L., et al., *Results of PROFILE 1029, a Phase III Comparison of First-Line Crizotinib versus Chemotherapy in East Asian Patients with ALK-Positive Advanced Non-Small Cell Lung Cancer.* J Thorac Oncol, 2018. **13**(10): p. 1539-1548.

53. Wu, Y.L., et al., *First-line erlotinib versus gemcitabine/cisplatin in patients with advanced EGFR mutation-positive non-small-cell lung cancer: analyses from the phase III, randomized, open-label, ENSURE study.* Ann Oncol, 2015. **26**(9): p. 1883-1889.

54. Yoh, K., et al., *A randomized, double-blind, phase II study of ramucirumab plus docetaxel vs placebo plus docetaxel in Japanese patients with stage IV non-small cell lung cancer after disease progression on platinum-based therapy.* Lung Cancer, 2016. **99**: p. 186-93.

55. Yoshioka, H., et al., *A randomized, double-blind, placebo-controlled, phase III trial of erlotinib with or without a c-Met inhibitor tivantinib (ARQ 197) in Asian patients with previously treated stage IIIB/IV nonsquamous nonsmall-cell lung cancer harboring wild-type epidermal growth factor receptor (ATTENTION study).* Ann Oncol, 2015. **26**(10): p. 2066-72.

56. Zhong, W.Z., et al., *Gefitinib versus vinorelbine plus cisplatin as adjuvant treatment for stage II-IIIA (N1-N2) EGFR-mutant NSCLC (ADJUVANT/CTONG1104): a randomised, open-label, phase 3 study.* Lancet Oncol, 2018. **19**(1): p. 139-148.

57. Zhou, C., et al., *BEYOND: A Randomized, Double-Blind, Placebo-Controlled, Multicenter, Phase III Study of First-Line Carboplatin/Paclitaxel Plus Bevacizumab or Placebo in Chinese Patients With Advanced or Recurrent Nonsquamous Non-Small-Cell Lung Cancer.* J Clin Oncol, 2015. **33**(19): p. 2197-204.
